# Genome-wide association study of common resistance to rust species in tetraploid wheat

**DOI:** 10.3389/fpls.2023.1290643

**Published:** 2024-01-03

**Authors:** Daniela Marone, Giovanni Laidò, Antonietta Saccomanno, Giuseppe Petruzzino, Cleber V. Giaretta Azevedo, Pasquale De Vita, Anna Maria Mastrangelo, Agata Gadaleta, Karim Ammar, Filippo M. Bassi, Meinan Wang, Xianming Chen, Diego Rubiales, Oadi Matny, Brian J. Steffenson, Nicola Pecchioni

**Affiliations:** ^1^ Centro di Ricerca Cerealicoltura e Colture Industriali, Consiglio per la Ricerca in Agricoltura e l'Analisi dell'Economia Agraria (CREA), Foggia, Italy; ^2^ Dipartimento di Scienze della Vita, Università di Modena e Reggio Emilia, Reggio Emilia, Italy; ^3^ Dipartimento di Scienze del Suolo, della Pianta e degli Alimenti (Di.S.S.P.A.), Università di Bari “Aldo Moro”, Bari, Italy; ^4^ International Maize and Wheat Improvement Centre (CIMMYT), Ciudad de México, Mexico; ^5^ International Center for Agricultural Research in the Dry Areas (ICARDA), Rabat, Morocco; ^6^ Department of Plant Pathology, Washington State University, Pullman, WA, United States; ^7^ Wheat Health, Genetics, and Quality Research Unit, United States Department of Agriculture - Agriculture Research Service (USDA-ARS), Pullman, WA, United States; ^8^ Institute for Sustainable Agriculture, Consejo Superior de Investigaciones Científicas (CSIC), Córdoba, Spain; ^9^ Department of Plant Pathology, University of Minnesota, St. Paul, MN, United States

**Keywords:** wheat rusts, *Puccinia* spp., tetraploid wheat, QTL, multi-location trials

## Abstract

Rusts of the genus *Puccinia* are wheat pathogens. Stem (black; Sr), leaf (brown; Lr), and stripe (yellow; Yr) rust, caused by *Puccinia graminis* f. sp. *tritici* (*Pgt*), *Puccinia triticina* (*Pt*), and *Puccinia striiformis* f. sp. *tritici* (*Pst*), can occur singularly or in mixed infections and pose a threat to wheat production globally in terms of the wide dispersal of their urediniospores. The development of durable resistant cultivars is the most sustainable method for controlling them. Many resistance genes have been identified, characterized, genetically mapped, and cloned; several quantitative trait loci (QTLs) for resistance have also been described. However, few studies have considered resistance to all three rust pathogens in a given germplasm. A genome-wide association study (GWAS) was carried out to identify loci associated with resistance to the three rusts in a collection of 230 inbred lines of tetraploid wheat (128 of which were *Triticum turgidum ssp. durum*) genotyped with SNPs. The wheat panel was phenotyped in the field and subjected to growth chamber experiments across different countries (USA, Mexico, Morocco, Italy, and Spain); then, a mixed linear model (MLM) GWAS was performed. In total, 9, 34, and 5 QTLs were identified in the A and B genomes for resistance to *Pgt*, *Pt*, and *Pst*, respectively, at both the seedling and adult plant stages. Only one QTL on chromosome 4A was found to be effective against all three rusts at the seedling stage. Six QTLs conferring resistance to two rust species at the adult plant stage were mapped: three on chromosome 1B and one each on 5B, 7A, and 7B. Fifteen QTLs conferring seedling resistance to two rusts were mapped: five on chromosome 2B, three on 7B, two each on 5B and 6A, and one each on 1B, 2A, and 7A. Most of the QTLs identified were specific for a single rust species or race of a species. Candidate genes were identified within the confidence intervals of a QTL conferring resistance against at least two rust species by using the annotations of the durum (cv. ‘Svevo’) and wild emmer wheat (‘Zavitan’) reference genomes. The 22 identified loci conferring resistance to two or three rust species may be useful for breeding new and potentially durable resistant wheat cultivars.

## 1 Introduction

Durum wheat (*Triticum turgidum* L. ssp. *durum*) is the most important cultivated tetraploid wheat in the world. Although durum is considered a minor crop because its global production is less than 5% of total wheat production, it is economically and nutritionally relevant because of its use for human consumption (e.g., pasta, couscous, bread, and bulgur), particularly in Mediterranean countries (Letta et al., 2014). Moreover, the tetraploid (AABB) wheat genomes show extensive similarities with the hexaploid (AABBDD) genomes of bread wheat (*Triticum aestivum* L.), and both crops often share the same pathogens. Foliar diseases caused by fungi, especially the three rusts (stem/black rust, leaf/brown rust, and stripe/yellow rust), can severely affect the yield and quality of the crop at a continental scale (Singh et al., 2011). Wheat rust pathogens have many common characteristics but also differ in key traits like their host ranges and conditions for infection, development, and survival. They can be effectively controlled by fungicide treatments; however, genetic resistance is the most economically and environmentally acceptable way to control them. Both qualitative and quantitative resistances have been described in durum wheat against the rusts. Qualitative resistance is usually conferred by one or a few genes (“R” genes) that have a large effect on the host reaction phenotype and are only effective against certain races of pathogens (*i.e.*, race-specific) (Yu et al., 2014). In many cases, these genes confer resistance from the seedling to the adult plant stage and are referred to as all-stage resistance (ASR) genes. By contrast, quantitative resistance is usually conferred by multiple genes or quantitative trait loci (QTL), with smaller effects on the host reaction phenotype. This type of resistance is generally effective against the spectrum of races within a pathogen and is therefore often considered as race non-specific. As the effect of these resistance loci is clearly manifested in adult plants, the resistance is referred to as adult plant resistance (APR) (Yu et al., 2014; Yu et al., 2017).

Stem rust, caused by *Puccinia graminis* Pers.:Pers. f. sp. *tritici* Erikss. & E. Henn. (*Pgt*), is a destructive disease of wheat in many parts of the world (Hodson et al., 2011). Over 60 (stem rust or *Sr*) genes and many QTLs against stem rust have been identified in wheat and its wild relatives (Saccomanno et al., 2018). Among these many described resistance loci, the most important race non-specific ones are *Sr57*, *Sr58*, *Sr55*, and *Sr2* (Juliana et al., 2017). Unfortunately, only a few (*i.e.*, *Sr55*, and *Sr57*) of these are still effective in different regions of Europe and North Africa due to the ability of the pathogen to overcome deployed resistance genes. Over the past decades, other virulent races of *Pgt* have been described in the United States and Sicily (TPMKC and TTTTF) (McVey et al., 2002; Bhattacharya, 2017), Uganda (TTKSK) (Jin et al., 2009; Rouse et al., 2014; Patpour et al., 2016), and Ethiopia (TRTTF, JRCQC, and TKTTF) (Olivera et al., 2012; Olivera et al., 2015).

Leaf rust, caused by *Puccina triticina* Erikss. (*Pt*), is the most widely distributed rust pathogen of wheat and is adapted to a wide range of environments. Approximately 100 leaf rust resistance genes and allelic forms have been identified and characterized in bread wheat, durum wheat, and diploid wheat species, and only eleven of them, *Lr1*, *Lr9*, *Lr10*, *Lr13*, *Lr14a*, *Lr21*, *Lr22a*, *Lr34*, *Lr42*, *Lr58* and *Lr67*, have been cloned, as recently reviewed by Mapuranga et al. (2022).

Stripe rust is caused by *Puccinia striiformis* Westend. f. sp. *tritici* Erikss. (*Pst*). Stripe rust infection may occur on the wheat plant from the time the first leaf emerges from the soil until maturity. Stripe rust has been found on every continent except Antarctica, with over 60 countries reporting outbreaks of the disease (Park, 2016). Ten stripe rust resistance genes (*Yr*) have been isolated so far: *Yr10*, *Yr18*, *Yr36*, *Yr46*, *Yr5/YrSP*, *Yr7*, *Yr15*, *Yr27*, *YrU1*, and *YrAS2388R* (Mapuranga et al., 2022). Among the cloned *Yr* genes, *Yr36* confers broad-spectrum resistance to races of *Pst* (Fu et al., 2009) and several confer multi-pathogen (partial) resistance against the three wheat rusts and powdery mildew (*Yr18*, *Lr34*, *Sr57*, *Pm38*, *Yr46*, *Lr67*, *Sr55*, and *Pm39*) (Krattinger et al., 2016).

Given the continuing threat of rust diseases, an ideal cultivar should be bred with durable resistance to all three of them; unfortunately, only a few identified genes, such as *Yr18*, *Lr34*, and *Sr57* and *Yr46*, *Lr67*, and *Sr55*, confer such broad-based resistance. In such a complex scenario of wheat/rust pathogen interactions of susceptibility and resistance, mapping QTLs can allow the detection of genes with major and minor effects and the identification of linked molecular markers that could be used for gene stacking in breeding programs to achieve more durable rust resistance (Soriano and Royo, 2015). The recent revolution in next-generation sequencing (NGS) technologies and the development of both low-cost and high-throughput SNP genotyping systems have promoted the rapid development of reliable markers for marker-assisted breeding in wheat. Traditionally, QTL mapping has been used to identify underlying genetic variation that co-segregates with a trait of interest using a biparental mapping population (Zhu et al., 2008). For example, Lin et al. (2018) reported a QTL for seedling and adult plant stripe rust resistance in a doubled-haploid population of durum wheat. This QTL for APR was mapped to chromosome 7B and validated in a breeding panel. In a recent study by Kumar et al. (2021), a stable major effect QTL was identified for stem rust resistance on the short arm of chromosome 6A in durum wheat. This QTL accounted for 71% of the variation for seedling resistance and up to 46% of the variation for field resistance.

Genome-wide association studies (GWAS) can dissect the genetic architecture of complex traits in natural populations, such as germplasm collections, often identifying a high number of QTLs with minor effects on phenotype together with those with major effects (Zhu et al., 2008; Hall et al., 2010). A GWAS conducted on a world collection of elite durum wheat accessions revealed a major effect locus for both seedling and adult plant stripe rust resistance (*Yrdurum-1BS.1*) (Liu et al., 2017). In a recent study by Kumar et al. (2020), a germplasm panel consisting of 483 spring bread wheat genotypes was phenotyped against all three rust diseases in greenhouse and field environments, and 25 genomic regions were found to be associated with resistance to at least two rusts. Of these, seven were associated with all the three rusts on chromosome groups 1 and 6 (A and B) along with 2B. Moreover, a meta-QTL analysis for multiple disease resistance (MDR) was carried out in hexaploid wheat and identified ten MQTLs involving all three rusts, overlapping with known R genes on different chromosomes (Pal et al., 2022). In this study, we conducted a large-scale association mapping study and a multi-location trial with the aim of identifying QTLs conferring seedling and adult plant resistance common to the three rusts of wheat in a single panel of 230 tetraploid genotypes, in both controlled and field conditions. Moreover, the availability of reference genome sequences of durum (cultivar ‘Svevo’) and wild emmer wheat (accession ‘Zavitan’) allowed the search for candidate genes in the physical region where QTLs for resistance to the rust pathogens were identified.

## 2 Materials and methods

### 2.1 Plant material

The germplasm collection used in this study consists of 230 inbred lines belonging to seven tetraploid wheat subspecies of *Triticum turgidum*, subdivided as follows: *Triticum turgidum* ssp. *durum* (128), ssp. *turanicum* (20), ssp. *turgidum* (19), ssp. *polonicum* (20), ssp. *carthlicum* (12), ssp. *dicoccum* (19), and ssp. *dicoccoides* (12). Laidò et al. (2013; 2014) provided a list and description of the genotypes (number/name, year of release, country, and pedigree) for each subspecies. Each accession was genotyped using the Illumina^®^ iSelect 90K wheat SNP assay (Wang et al., 2014). The resulting dataset was filtered using the following criteria: (1) markers showing residual heterozygosity were entered as missing values; (2) markers with less than 10% missing data and accessions with less than 20% missing data were retained; and (3) markers with a minor allele frequency (MAF) greater than 10% were retained. The genotypic analyses were finally carried out on three distinct subsamples as described in Laidò et al. (2013; 2014) and Saccomanno et al. (2018): the whole collection (230 genotypes, WC), the durum subsample (127 genotypes, durum), and the Q2 group (98 genotypes, Q2), which mainly contains wild and domesticated accessions of tetraploid wheat other than durum. The remaining five genotypes (Russello, ssp. durum; PI 278350, ssp. *turanicum*; PI 361757 and PI 208911, ssp. *polonicum*; PI 352324, ssp. *dicoccoides*) were excluded because they had >20% missing markers.

### 2.2 Phenotypic evaluations

**Table 1** summarizes all the details of the phenotypic evaluations of the tetraploid collection in terms of the reactions to the three rust species at the seedling and adult plant stages, challenged with either single virulent isolates in controlled conditions or natural pathogen populations in field experiments from 2015 to 2016 (Morocco, Spain, Italy, Mexico, and USA). The phenotypic data were collected by the participating institutions as disease severity (DS) and/or infection type (IT) data. Field experiments conducted by CREA, CSIC, and CIMMYT employed a randomized complete block design with two replicates, whereas those conducted by USDA-ARS, the University of Minnesota, ICARDA, and CREA in 2016 were organized according to the model replicated check. In particular, using as controls, the susceptible wheat line “PS279” in the USDA-ARS experiments with stripe rust, “Rusty” in the University of Minnesota experiments with stem rust, and “Pedroso” for the ICARDA and CREA experiments with stripe and leaf rust. The accessions in all field experiments were sown in 1.0 to 1.5 m long rows. Rust disease severity (DS) was assessed visually on the stem and leaf sheath, two times a week, as the percentage area (0, 5, 10, 20, 40, 60, 80, or 100%) covered by pustules, using the modified Cobb scale for stripe rust and leaf rust (Peterson et al., 1948). Stem rust severity was assessed on a 0–100% scale using the diagrammatic scale of James (1971).

**Table 1:** List of phenotyping trials conducted on the tetraploid wheat collection at the adult (field) and seedling (controlled environment) stages at different locations and in different conditions.

Plant stage	Institution	Infection	Environment	Coordinates	Rust	Race	Sowing season
**Adult**	USDA-ARS	Natural	Pullman, USA	*46° 43’ N, 117° 10′ W*	*Yr*	Natural population (1)	Spring 2015
	USDA-ARS	Natural	Pullman, USA		*Yr*	Natural population (2)	Autumn 2015
	USDA-ARS	Natural	Mt. Vernon, USA	*48° 25′ N, 122° 19′ W*	*Yr*	Natural population (3)	Spring 2015
	ICARDA	Natural	Marchouch, Morocco	*33° 56′ N, 6° 69′ W*	*Yr*	Natural population (4)	Autumn 2015
	ICARDA	Natural	Marchouch, Morocco		*Lr*	Natural population (5)	Autumn 2015
	ICARDA	Natural	Allal Tazi, Morocco	*34° 52′ N, 6° 31′ W*	*Lr*	Natural population (6)	Autumn 2016
	CREA	Natural	Foggia, Italy	*41° 28′ N, 15° 32′ E*	*Yr*	Natural population (7)	Winter 2015
	CREA	Natural	Foggia, Italy		*Lr*	Natural population (8)	Winter 2015
	CREA	Natural	Foggia, Italy		*Yr*	Natural population (9)	Winter 2016
	Univ. Minnesota	Artificial	Saint Paul, USA	*44° 58′ N, 93° 14′ W*	*Sr*	Mix of six races: QFCSC, QTHJC, MCCFC, RCRSC, RKQQC, and TPMKC (10)	Spring 2015
	CSIC	Artificial	Cordoba, Spain	*37° 88′ N, 47° 79′ W*	*Lr*	CONDESA (11)	Autumn 2015
	CIMMYT	Artificial	Toluca, Mexico	*19° 28′ N, 99° 65′ W*	*Yr*	MX14-191 (12)	Summer 2016
	CIMMYT	Artificial	El Batan; Mexico	*19° 52′ N, 98° 85′ W*	*Lr*	BBG-BP (13)	Summer 2016
	CIMMYT	Artificial	El Batan, Mexico		*Yr*	MX14-191 (14)	Summer 2016
	CIMMYT	Artificial	Obregon, Mexico	*27° 48′ N, 109° 93′ W*	*Lr*	BBG-BP (15)	Autumn 2016
**Seedling**	Univ. Minnesota	Artificial	Greenhouse	*44° 58′ N, 93° 14′ W*	*Sr*	TTTTF (16)	–
	Univ. Minnesota	Artificial	Greenhouse		*Sr*	TPMKC (17)	–
	Univ. Minnesota	Artificial	Greenhouse		*Sr*	TRTTF (18)	–
	Univ. Minnesota	Artificial	Greenhouse		*Sr*	JRCQC (19)	–
	Univ. Minnesota	Artificial	Greenhouse		*Sr*	TKTTF (20)	–
	Univ. Minnesota	Artificial	Greenhouse		*Sr*	TTKSK (21)	–
	Univ. Minnesota	Artificial	Growth chamber		*Yr*	PSTv-14 (22)	–
	Univ. Minnesota	Artificial	Growth chamber		*Yr*	PSTv-37 (23)	–
	Univ. Minnesota	Artificial	Growth chamber		*Yr*	PSTv-40 (24)	–
	Univ. Minnesota	Artificial	Greenhouse		*Lr*	Spain 5-2 (25)	–
	Univ. Minnesota	Artificial	Greenhouse		*Lr*	PSB14 (26)	–
	CSIC	Artificial	Growth chamber	*37° 88′ N, 47° 79′ W*	*Lr*	CONIL (27)	–
	CSIC	Artificial	Growth chamber		*Lr*	CONDESA (28)	–

Rust severity (DS) was assessed in adult plants in the field experiments and infection types (IT) in seedlings in controlled environments. When plants were challenged with specific isolates, their name was reported. Identification numbers of each experiment are inserted in brackets, which were conducted with natural populations, a mix of races, and single isolates of the three pathogens.

Lr, leaf rust; Yr, stripe rust; Sr, stem rust.

For the seedling experiments, at the University of Minnesota, inoculations for the reaction to *Pgt* were conducted in a standard GH7 greenhouse for the domestic races of stem rust TTTTF and TPMKC; the exotic races TRTTF, JRCQC, TKTTF, and TTKSK were inoculated in a greenhouse at the Biosafety Level-3 (BSL-3) Containment Facility of the Saint Paul campus. As for the reaction to Pst, inoculations for races PSTv-14, PSTv-37, and PSTv-40 were undertaken in a Biosafety Level-2 (BLS-2) growth chamber, whereas inoculations to evaluate Pt with races PSB14 and Spain 5-2 were conducted in a BSL-3 growth chamber.

In the leaf rust experiments carried out in the greenhouse at CSIC, the isolates CONDESA and CONIL were used. The *Pst* races used at the University of Minnesota were provided by USDA-ARS and included PSTv-37, a predominant race in the United States, PSTv-14 and PSTv-40, which are mainly found in the Pacific Northwest and California, and Pt isolates PSB14 and Spain 5-2, which are virulent races of Italian and Spanish origins. The seedling experiments were conducted using a completely randomized design with two replicates (three plants for each replicate) at the University of Minnesota and three replicates (five plants for each replicate) at CSIC. Any accession exhibiting variable reactions across replicates was tested again in an additional experiment. The wheat genotype Rusty was used as a susceptible control for the stem rust seedling experiments. For the stripe rust experiments, Morocco and PS279 were used as susceptible checks and Madsen as the resistant check. For the leaf rust experiments, Tc Lr37 and Little Club were used as the resistant and susceptible controls, respectively (Steffenson et al., 2017). In the greenhouse, stem rust ITs were assessed using the 0–4 scale of Stakman et al. (1962), whereas for leaf rust, the scale described by Long and Kolmer (1989) was used. In both scales, ITs from 0 to 2 represent resistant reactions and those from 3 to 4 represent susceptible reactions. Stripe rust infection types were scored using the 0-9 scale described by Chen (2013), in which ITs from 0 to 6 represent resistant reactions and those from 7 to 9 represent susceptible reactions. To meet the data format required for association mapping analysis, the raw seedling IT data were converted to a 0–10 linear disease scale for stem rust and a 0-9 scale for stripe and leaf rust. In cases in which lines exhibited a heterogeneous reaction, only the most prevalent IT was used in the analysis.

### 2.3 Statistical analysis

Descriptive statistics and analysis of variance (ANOVA) were performed for each experiment. The genotype means were compared using the least significant difference (LSD) at a 5% probability level. Additionally, genetic variance (σ^2^G) and broad-sense heritability (*H_2_*) were estimated. Statistical analyses were conducted using the statistical computation software R (http://www.r-project.org). The experiments were carried out using the following design settings: 1) a randomized complete block design according to the model Yij= μ+ G_i+ B_j+ ϵ_ij, where Yij is the value of the characteristic of the i-th response in the i-th block, μ is the general mean, Gi is the effect of the i-th RSI (i = 1,2,…, g), Bj is the effect of the j-th block (j = 1,2,…, r), and ϵij is the random error; and 2) a replicated check design according to the equation Yij= μ+ T_i+ ϵ_ij, where Yij is the value of the characteristic of the i-th response, μ is the general mean, Ti is the effect of the i-th replicated check, and ϵij is the random error.

### 2.4 Population structure, linkage disequilibrium, and association mapping analysis

After filtering the SNP dataset for marker residual heterozygosity, missing data, and alleles with <10% MAF, 17,678, 12,225, and 19,191 polymorphic SNP markers were retained for the whole collection, durum subsample, and Q2 group, respectively; these markers were then used for population structure and association mapping analyses. Using the high-density consensus tetraploid wheat map reported by Maccaferri et al. (2015), 16,425, 12,194, and 16,744 mapped SNP markers were used for linkage disequilibrium analysis for the whole collection, durum subsample and Q2 group, respectively. Correlated markers distributed every 1, 2, and 10 cM intervals on the genome were selected to evaluate population structure. Population structure was also assessed based on uncorrelated SNPs using the tagger function r^2^ = 0.5, 0.8, and 1 in Haploview 4.2 (Barrett et al., 2005).

Population structures, for the whole collection, durum subsample, and Q2 group separately, were analyzed using a Bayesian model-based clustering approach and principal component analysis (PCA). The SNP data were processed using the STRUCTURE software package v.2.3.4 (Pritchard et al., 2000) (http://pritch.bsd.uchicago.edu/structure.html); the number of subgroups (K) was estimated after 20 independent runs for each K (from 2 to 20), as reported by Saccomanno et al. (2018). A genotype was considered to belong to a group if its membership coefficient was ≥0.50 (Royo et al., 2010).

TASSEL software (ver. 5.2.18) was used to calculate the LD (allele frequency correlation, r^2^) between all marker pairs in the three groups. The significance of the pairwise LD (*P* values) was computed using 1,000 permutations. LD statistics were calculated individually for each chromosome and subsequently aggregated over all the chromosomes of the A and B genomes. LD was calculated as reported by Saccomanno et al. (2018). The map distance at which LD fell below the r^2^ threshold of 0.3 was used to define the confidence intervals of the QTLs detected in this study (Ardlie et al., 2002).

For GWAS, TASSEL (ver. 5.2.18) was used to conduct mixed linear model (MLM) analyses. Marker-trait associations (MTAs) were determined by two statistical models: MLM+K+Q and MLM+K. The loci considered for association mapping were characterized by the frequency of the rarest allele >0.1. The trimmed marker dataset was used to generate a marker similarity matrix containing all the lines (K matrix); once the matrix was calculated, the numbers were rescaled between 0 and 2 (Papa et al., 2007). The kinship matrix was implemented in TASSEL for the MLM based on the kinship matrix (MLM+K) and the MLM based on both the K matrix and Q matrix (MLM+K+Q). In addition to the genotypic and phenotypic data, the Q matrix was integrated as a covariate to correct for the effects of population substructure, and the kinship matrix (K) was used to correct the family structure effect.

The critical *P*-value for the assessment of the significance of marker-trait associations (MTAs) was calculated based on a false discovery rate (FDR) of 0.05 (Mosig et al., 2001). The algorithm by Benjamini and Hochberg (1995) was used for FDR correction; FDR and Bonferroni corrections were applied for *p*-values obtained for the MLM. The genetic positions of MTAs were compared with those previously reported in the literature based on common markers between genetic maps.

### 2.5 The search for candidate genes

For candidate gene identification, the nucleotide sequences of each QTL peak marker, as well as markers included in the respective confidence interval for each QTL, according to genome-wide LD decay, were used as queries in a BLASTN search (threshold, E-10) against the gene sets of the *T. dicoccoides* accession ‘Zavitan’ (Avni et al., 2017) and the durum wheat cultivar ‘Svevo’ (Maccaferri et al., 2019). All genes occurring in the confidence intervals were thus retrieved along with their functional annotations.

## 3 Results

### 3.1 Phenotypic evaluations and the results of statistical analyses

In the tetraploid wheat collection, a high level of variability was recorded for the reaction to the three rust species in all experiments, except in the field experiment at Marchouch, where very low levels of leaf rust infection were observed (**Supplementary Tables 1, 2**). High heritability values were observed, indicating a significant weight of the genetic component on the traits (**Supplementary Tables 1, 2**). In the field experiments, broad sense heritability ranged from 62.0% to 90.1% for the stripe rust reaction, from 75.8% to 93.6% for leaf rust, and 91.5% for stem rust (**Supplementary Table 1**). For the seedling experiments, heritability values were all above 91%, except those in response to the *Pt* races PSB14 and SPAIN52 (88.8% and 77.4%, respectively; **Supplementary Table 2**). Statistically significant differences were found across genotypes for all experiments conducted in this study, with the most significant p-values recorded for the experiments in controlled conditions (**Supplementary Tables 3, 4**). The frequency distributions of DS and IT at the adult plant and seedling stages, respectively, suggested a complex genetic control for these traits (**Supplementary Figures 1-6**). A prevalence of susceptible genotypes was observed in the seedling experiments against all *Pt* races, all *Pst* races with the exception of PSTv-14, and against all *Pgt* races except JRCQC (**Supplementary Figures 4-7**). If the IT data of the germplasm are divided into subspecies, some differences in the proportion of resistant *vs*. susceptible accessions were noted, but this also depended on the rust isolate challenged (**Supplementary Figures 8-10**). The wild emmer group was in general characterized by a higher proportion of susceptibility; moreover, very limited phenotypic variation was observed in response to any of the races of the three rusts, the exceptions being with the *Pst* race PSB14 and the *Pgt* race TKTTF. Other germplasm groups exhibited a wider variation of reaction types. For example, durum wheat and domesticated emmer (*T. turgidum* ssp. *dicoccum*) had a higher number of resistant to highly resistant genotypes, although the two samples were unbalanced in their number of accessions. The durum wheat group was the most variable in response to all tested races of rust pathogens, the exception being the *Pt* isolate CONDESA, against which a higher number of resistant genotypes were found among the domesticated emmers *vs*. durums. Correlation and PCA-Biplot analysis generally identified more positive than negative correlations, with the highest positive being found as expected between the reactions to isolates of the same pathogen species. However, within each rust species, positive correlations were also found between controlled and field experiments (**Supplementary Figures 11-14**). Interestingly, negative correlations were recorded among reactions to the *Pgt* (stem rust) isolates and leaf rust in field experiments (**Supplementary Figure 11**). If the selection of genotypes is undertaken on the basis of their resistance spectrum against each single isolate of the three rust species, the main observations are as follows. The durum subgroup and the *T. turgidum* ssp. *polonicum* genotypes showed, in general, the highest level of resistance to the three races of stripe rust (**Supplementary Figure 9**). In particular, 13 durum varieties and 5 accessions of ssp. *polonicum* were identified as resistant to all races (**Supplementary Figures 7, 9**). The durum wheat cultivars Altar 84, Granizo, and Grazia showed strong resistance to all *Pgt* and *Pst* races tested. Additionally, the accession PI 366117 of *T. turgidum* ssp. *polonicum* was notable for its resistance to all *Pgt* and *Pst* races, as well as to the *Pt* race PSB14. *Pt* races CONIL and CONDESA were highly virulent as a high degree of susceptibility was observed across all the subspecies (**Supplementary Figure 10**). With respect to individual accessions within a subspecies, the genotype MG5300/1 of *T. turgidum* ssp. *dicoccum* was identified as resistant to the four races of *Pt*, and the durum wheat cultivars Saragolla and Giotto were resistant to the CONIL race.

### 3.2 Population structure and linkage disequilibrium

The population structure was analyzed using a Bayesian approach, as implemented in the STRUCTURE software, following Evanno et al. (2005). The results generated maximum ΔK values occurring at K=3, K=4, and K=6 for the whole collection, at K=5 and K=6 for the durum subsample, and at K=5 and K=8 for the Q2 group considering one marker every 1 cM. In the whole collection, durum subsample, and Q2 group for one marker every 2 cM, maximum ΔK was found at K=3, K=4, and K=8, at K=6, and at K=4, K=6, and K=8, respectively. When one marker every 10 cM was considered, the maximum ΔK values found were at K=3 and K=6 for the whole collection, at K=4 and K=5 for the durum subsample, and at K=3, K=5, and K=7 for the Q2 group. For non-correlated SNP markers, the results obtained, considering r^2^ at 0.5, 0.8, and 1, were similar; in particular, K=3 for the whole collection, K=6 for the durum subsample, and K=5 and K=6 for Q2 group.

Linkage disequilibrium analysis was performed for the whole collection, durum subsample, and Q2 group by using the SNP markers aligned on the consensus map in Maccaferri et al. (2015). The plots of the LD estimates (r^2^) as a function of genetic distance (cM) indicated a clear decay of LD with genetic distance and suggested LD was dependent on population structure. The point at which the LOESS curve intercepted the critical r^2^ was determined as the average LD decay of the population. The scatterplot of the distributions of the r2 values as a function of the genetic distance between intra-chromosomal pairs showed LD decays for the whole collection, durum subsample, and Q2 group of approximately 9 cM, 20 cM, and 1 cM, respectively (**Table 2**). Based on these criteria, the LD decays for the individual chromosomes ranged from ~1 cM for chromosome 3B to ~13 cM for chromosome 2A (**Supplementary Material 2.1**) for the whole collection, from ~9 cM for chromosomes 6B and 4B to ~23 cM for chromosome 3B (**Supplementary Material 3.2**) for the durum subsample, and from ~1 cM for several chromosomes to ~7 cM for chromosomes 2A and 5A (**Supplementary Material 4.1**) for the Q2 group.

**Table 2:** Mean linkage disequilibrium (LD) decay between intra-chromosomal pairs for the whole collection, durum subsample, and Q2 group for single chromosomes and the whole genome.

Chromosome	Whole collection	Durum subsample	Q2 group
1A	5 cM (948)	14 cM (623)	4 cM (1,144)
1B	8 cM (1,663)	12 cM (1,273)	1 cM (1,620)
2A	13 cM (1,129)	19 cM (832)	7 cM (1,120)
2B	6 cM (1,710)	11 cM (1,206)	2 cM (1,743)
3A	9 cM (925)	12 cM (644)	5 cM (952)
3B	1 cM (1,218)	23 cM (753)	1 cM (1,322)
4A	5 cM (781)	11 cM (893)	1 cM (791)
4B	7 cM (915)	9 cM (726)	3 cM (867)
5A	10 cM (951)	16 cM (613)	7 cM (1,027)
5B	5 cM (1,346)	18 cM (923)	1 cM (1,364)
6A	10 cM (1,051)	12 cM (770)	4 cM (1,046)
6B	7 cM (1,223)	9 cM (940)	1 cM (1,207)
7A	12 cM (1,285)	19 cM (1,001)	1 cM (1,270)
7B	10 cM (1,280)	15 cM (997)	1 cM (1,271)
**Whole genome**	**9cM (16,425)**	**20cM (12,194)**	**1 cM (16,744)**

For each chromosome, the number of mapped SNP markers is reported in brackets.

### 3.3 Resistance QTLs

Association mapping analyses were conducted using MLM + K as the best model for the subsamples used in this study (**Supplementary Material 5**, a to f). Analyses were carried out separately for each rust species on the three different subsamples, taking into account all environments tested and the plant growth stages. In **Table 3A**, the number of QTLs defined by at least two significant closely linked markers at a FDR <0.05 is reported for each rust species at both growth stages for each subsample. The highest number of QTLs (26) was found for leaf rust resistance at the adult plant stage, 23 of which were identified in the whole collection.

**Table 3:** Results of association mapping analyses carried out separately for each rust pathogen.

A							
Subsample	Number of QTL (no. of MTAs)						Total
	Stem rust		Stripe rust		Leaf rust		
	Seedling	Adult	Seedling	Adult	Seedling	Adult	
Whole collection	–	–	–	1 (5)	6 (31)	23 (165)	30
Durum	1 (11)	–	–	–	–	–	1
Q2	5 (22)	2 (15)	1 (3)	2 (10)	–	–	10
WC/Q2	–	–	–	–	–	2 (26)	2
WC/durum	1 (24)	–	–	–	2 (12)	1 (16)	4
WC/Q2/durum	–	–	1 (8)	–	–	–	1
Total	7 (57)	2 (15)	2 (11)	3 (15)	8 (43)	26 (207)	48
B							
Subsample	Number of single MTAs						Total
	Stem Rust		Stripe Rust		Leaf Rust		
	Seedling	Adult	Seedling	Adult	Seedling	Adult	
Whole collection	1	–	–	–	19	3	23
Durum	1	–	–	–	14	1	16
Q2	7	3	2		–	–	13
WC/Q2	–	–	–	–	–	–	0
WC/durum	1	–	–	–	3	2	6
WC/Q2/durum	–	–	–	–	–	–	0
Total	10	3	2	0	36	6	57

A) The number of QTLs identified by at least two closely linked markers, reported for each rust pathogen at both growth stages and for each subsample. The number of MTAs that define the QTL are shown in brackets. QTLs found in common between subsamples are also indicated. B) Single MTAs at a FDR <0.01 identified for each rust/subsample are reported.

With respect to single MTAs for resistance to each rust species, taking into account a high level of significance (FDR<0.01) to avoid false positives, a total of 57 MTAs were identified. Of these, the majority of them (36) were associated with seedling resistance to leaf rust (**Table 3B**).

**Figure 1:**
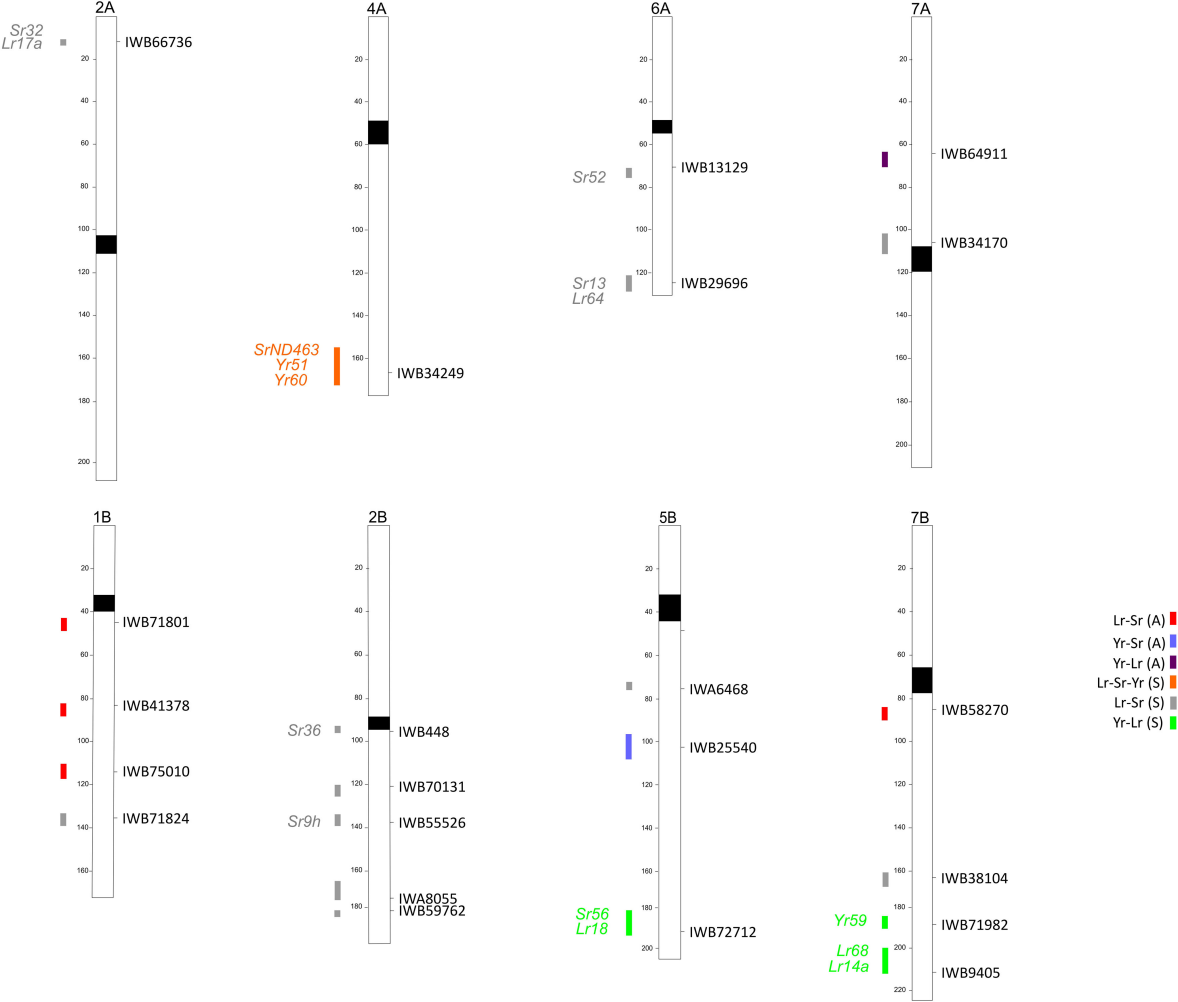
Schematic representation of durum wheat chromosomes based on the durum consensus linkage map (Maccaferri et al., 2015) with map positions of QTLs for rust resistance identified. QTL are identified by confidence intervals on the left side and the QTL tagging markers on the right side of chromosomes.

**Tables 4** and **5**, respectively, and **Figure 1** show the co-mapping QTLs of resistance to more than one rust species identified at the adult plant and seedling stages. The haplotype peak, defined as the marker with the lowest *p-value* among those of the single QTL identified by any phenotypic study, is the only marker reported, together with its functional annotation. In particular, six different QTLs for resistance were found in common to the different rusts at the adult plant stage, of which four were for resistance to both leaf and stem rust; the remaining two (one each) were for resistance to stem and stripe rust, and to stripe and leaf rust (**Table 4**; **Figure 1**). As adult plant resistance has been detected with artificial and natural inoculations, we found a higher number of associations under natural infection, particularly from the ICARDA site for testing resistance to leaf rust (**Supplementary Material 6**).

**Table 4:** Quantitative trait loci (QTL) conferring adult plant resistance to more than one rust pathogen.

Chr.	QTL no.	QTL name	Rust diseases (experiments)	Haplotype peak (functional annotation)	Position (cM)	*P* value	Subsample	R^2^ (%)	CI (cM)	References
1B	1	QLrSr(A)-1B.1	Lr(5)-**Sr**(10)	IWB71801 (shikimate kinase like 2)	43.5	3.30E-05	WC	9	4.1	Yr (Liu et al., 2017)
	2	QLrSr(A)-1B.2	Lr(5)-**Sr**(10)	IWB41378 (na)	84.5	6.73E-06	WC	12	3.8	Sr (Letta et al., 2013; Letta et al., 2014) Yr (Liu et al., 2017)
	3	QLrSr(A)-1B.3	Lr(5)-**Sr**(10)	IWB75010 (Imidazole glycerol phosphate synthase subunit HisF)	115.4	3.28E-09	WC	20	6.0	Sr (Letta et al., 2013) Yr (Liu et al., 2017)
5B	4	QYrSr(A)-5B	Yr(1-3-7)-**Sr**(10)	IWB25540 (UTP-glucose-1-phosphate uridylyltransferase)	103.8	2.42E-09	WC	17	9.2	–
7A	5	QLr-Yr(A)-7A	Lr(5)-**Yr**(3)	IWB64911 (Post-GPI attachment to proteins factor 3)	65.5	1.07E-07	Q2	50	5.2	Lr (Aoun et al., 2016) Sr (Haile et al., 2012; Letta et al., 2013; Aoun et al., 2019)
7B	6	QLrSr(A)-7B	Lr(5)-**Sr**(10)	IWB58270 (Omega-hexatoxin-Asp2b)	86.4	6.00E-05	WC/Q2	20	4.1	Sr (Letta et al., 2013)

In the column “Rust diseases (experiments)” the diseases controlled by the QTL are reported, whereas the bold letters indicate the rust pathogen against which the QTL is primarily acting. The number of the experiments referred to in **Table 1** are shown in brackets. The haplotype peak is the MTA with the lowest P-value in the QTL and is referred to the rust reported in bold; the annotation of the related gene is reported in the brackets, if any was identified. The genetic position in the chromosome, the P-value, the subsample in which the QTLs were identified, and the R^2^ are referred to the haplotype peak. CI indicates the confidence interval of the QTL as the map distance at which LD fell below the r^2^ threshold of 0.3. The “References” column contains studies reporting previously identified resistance genes and QTLs against these pathogens. Yr, stripe rust; Lr, leaf rust; Sr, stem rust.

**Table 5:** Quantitative trait loci (QTLs) conferring seedling resistance to more than one rust pathogen.

Chr.	QTL no.	QTL name	Rusts (experiments)	Haplotype peak (functional annotation)	Position (cM)	*P* value	Subsample	R^2^ (%)	CI (cM)	References
1B	7	QLrSr(S)-1B	Lr(27)/Sr(19)	IWB71824 (Subtilisin-like protease)	136.6	2.49E-10	Q2	35	4.1	Sr (Letta et al., 2013; Laidò et al., 2015) Yr (Liu et al., 2017)
2A	8	QLrSr(S)-2A	**Lr**(25-27)/Sr(21)	IWB66736 (NBS-LRR-like resistance protein)	11.2	1.97E-12	Durum	36	0.0	Lr (Aoun et al., 2016) Sr (Letta et al., 2014) Yr (Liu et al., 2017)
2B	9	QLrSr(S)-2B.1	**Lr**(27)/Sr(18)	IWB448 (ChaperoneproteindnaJ)	95.2	1.73E-08	Q2	29	0.3	Lr (Aoun et al., 2016) Sr (Saccomanno et al., 2018) Yr (Naz et al., 2012)
	10	QLrSr(S)-2B.2	**Lr**(27)/Sr(18-19)	IWB70131 (na)	120.2	1.79E-05	Durum	26	4.8	Sr (Aoun et al., 2019)
	11	QLrSr(S)-2B.3	**Lr**(27)/Sr(18-19)	IWB55526 (NBS-LRR disease resistance protein-like protein)	137.9	2.61E-09	WC/durum	17	4.0	Sr (Letta et al., 2013; Saccomanno et al., 2018; Saini et al., 2018) Yr (Liu et al., 2017)
	12	QLrSr(S)-2B.4	**Lr**(27)/Sr(20)	IWA8055 (Callose synthase-like protein)	174.6	2.38E-08	Durum	25	8.6	Sr (Letta et al., 2013; Laidò et al., 2015) Yr (Liu et al., 2017)
	13	QLrSr(S)-2B.5	**Lr**(27-28)/Sr(16)	IWB59762 (Cleavage and polyadenylation specificity factor subunit 5)	182.7	5.13E-18	WC	31	3.0	–
4A	14	QLrYrSr(S)-4A	**Lr**(27-28)/Yr(24)/**Sr**(19-20)	IWB34249 (Acetyltransferase component of pyruvate dehydrogenase complex)	168.6	1.13E-17	WC/durum/Q2	30	16.9	Sr (Letta et al., 2013; Saccomanno et al., 2018; Saini et al., 2018) Yr (Liu et al., 2017)
5B	15	QLrSr(S)-5B.1	Lr(28)/**Sr**(19-21)	IWA6468 (ADP-ribosylation factor GTPase-activating protein)	75.4	1.09E-07	WC	14	1.1	Sr (Saccomanno et al., 2018) Yr (Liu et al., 2017)
	16	QLrYr(S)-5B	**Lr**(27-28)/Yr(23)	IWB72712 (na)	192.3	5.62E-15	WC	27	9.8	Sr (Letta et al., 2013) Yr (Liu et al., 2017)
6A	17	QLrSr(S)-6A.1	Lr(28)/**Sr**(18)	IWB13129 (na)	71.8	3.13E-07	WC	13	0.6	Lr (Aoun et al., 2016) Sr (Haile et al., 2012; Saccomanno et al., 2018)
	18	QLrSr(S)-6A.2	Lr(27-28)/**Sr**(21)	IWB29696 (na)	126.7	1.82E-07	Durum	24	7.9	Lr (Aoun et al., 2016) Sr (Letta et al., 2013; Saccomanno et al., 2018; Saini et al., 2018)
7A	19	QLrSr(S)-7A	Lr(28)/**Sr**(19)	IWB34170 (Pyrophosphate-fructose 6-phosphate 1-phosphotransferase subunit beta)	107.6	1.39E-06	Q2	23	10.3	Sr (Haile et al., 2012) Yr (Liu et al., 2017)
7B	20	QLrSr(S)-7B	Lr(28)/**Sr**(19)	IWB38104 (Receptor-like protein kinase)	164.4	6.89E-11	Q2	37	5.7	Sr (Letta et al., 2013; Saccomanno et al., 2018)
	21	QLrYr(S)-7B.1	Lr(28)/**Yr**(23-24)	IWB71982 (Mediator of RNA polymerase II transcription subunit 15a)	187.5	1.21E-24	WC/durum	39	5.0	Lr (Marone et al., 2009) Yr (Liu et al., 2017)
	22	QLrYr(S)-7B.2	**Lr**(26-28)/Yr(23-24)	IWB9405 (Rp1-like protein)	211.5	1.22E-09	Durum	29	12.2	Lr (Maccaferri et al., 2008; Marone et al., 2009; Terracciano et al., 2013; Aoun et al., 2016) Sr (Haile et al., 2012; Kthiri et al., 2018) Yr (Singh et al., 2013)

The haplotype peak is the marker-trait association (MTA) with the lower P-value in the region; the annotation of the related candidate gene is reported in brackets, if any was identified (na, none). The genetic position in the chromosome, the P-value, and the subsample in which the QTLs were identified are referred to the haplotype peak. The R^2^ is the highest value of the percentage of the phenotypic variation for resistance against a single rust disease identified for each QTL. CI indicates the confidence interval of the QTL as the map distance at which LD falls below the r^2^ threshold of 0.3. In the column “Rusts (experiments)”, the bold letters indicate the rust pathogen against which the QTL is primarily acting; the experiments in which the QTL was identified, numbered in accordance with **Table 1**, are shown in brackets. The “References” column shows reports of previously identified resistance genes and QTLs against these pathogens. Yr, stripe rust; Lr, leaf rust; Sr, stem rust.

Among the four Sr-Lr QTLs, only the locus on 7B was confirmed in two different subsamples (WC and Q2), whereas the other three associations were found in the WC only. The QTL on chromosome 5B, involved in stem and stripe rust resistance, was identified in the WC. Finally, the resistance QTL common to leaf and stripe rust on 7A was found in the Q2 subsample. With respect to the percentage of explained variability (R^2^) for the QTL harboring leaf rust and stem rust MTAs, the highest R^2^ values were always found for stem rust resistance. The same was true for resistance QTLs to *Pst* and *Pgt* on 5B. On the contrary, regarding the co-mapping of resistance QTLs to leaf and stripe rust on 7A, the one targeting the latter notably explained 50% of the trait variation observed, which was more than that for leaf rust (23%) (**Table 4**; **Supplementary Material 6**).

Sixteen QTLs were involved in resistance to more than one rust species at the seedling stage and were dispersed across the genome, except a couple homoeologous QTLs on 3A and 3B and on chromosomes 1A, 4B, 5A, and 6B (**Table 5**; **Figure 1**). Fifteen of them conferred resistance to two species, and in particular 12 to Sr-Lr and 3 to Lr-Yr. Therefore, as in the case of the resistance QTL active at the adult plant stage, the most common co-mapping QTLs were those for resistance to leaf and stem rust. No loci conferring resistance against both stem rust and stripe rust were identified. Finally, and most interestingly, a region on the long arm of chromosome 4A, with its peak marker IWB34249 identified in all three datasets, conferred resistance to all three rusts (**Table 5**; **Supplementary Material 6**).

Associations were highly significant for the mapped QTLs, particularly for the one common to the three pathogens mapped on 4A (Lr-Yr-Sr, *P*-value 1.13E-17) and two other QTLs tagging two out of three species on 7B (Lr-Yr, P-value 1.21E-24) and 2B (Lr-Sr, *P*-value 5.13E-18). All loci were identified by analyzing a single subsample of the collection, with the exception of Lr-Sr on 2B, Lr-Yr-Sr on 4A, and Lr-Sr on 7B, in which the associations were confirmed in two different subsamples (**Table 5**). The QTL conferring resistance to the three rust species on 4A contributed greatly to the resistance against leaf and stem rust by explaining nearly 30% of the variability observed for each species, whereas it controlled 19% of the phenotypic variation for stripe rust. Among the QTLs conferring resistance to two pathogens, those on 2A and 2B contributed significantly to leaf rust resistance, explaining more than 30% of the phenotypic variation. They were mapped in the durum subsample, with the only exception being the QTL at 95.2 cM on 2B, which was identified in the Q2 group (**Table 5**; **Supplementary Material 6**). On the contrary, the QTLs on 1B, 5B, 6A, 7A, and 7B (165 cM) all explained the resistance to stem rust and were all significant in the Q2 subsample, except for the one on 5B identified in the whole collection. As for the common regions of resistance against leaf rust and stripe rust, three regions were detected, two on 5B and one on 7B (at 211 cM), contributing more to leaf than to stripe rust resistance and mapping in the WC and durum subsamples, respectively. The third region on 7B (187 cM) was identified in the durum subsample, and it notably explained approximately half (49%) of the observed variation for resistance against stripe rust.

### 3.4 Defense-related candidate genes within the resistance QTL effective against two and three rust species

The sequence of the peak QTL markers associated with resistance to multiple rust species, as well as the markers of the QTL confidence interval, were used to search for candidate genes. The results are summarized in **Table 6**, with additional detailed information presented in the **Supplementary Materials 8, 9**.

**Table 6:** Summary of the present annotated functional categories in each quantitative trait locus genomic region.

				Zavitan genome				Svevo genome			
	QTL in common for two and three diseases	Chr.	Genetic interval (cM)	Physical interval (Mb)	Start (bp)	End (bp)	Functional categories annotated	Physical interval (Mb)	Start (bp)	End (bp)	Functional categories annotated
ADULT STAGE	Lr-Sr	1B	4.1	23.3	388,767,287	412,082,481	104	23.9	375,381,007	399,258,925	313
	Lr-Sr	1B	3.8	6.3	566,536,184	572,833,358	38	6.5	554,341,414	560,850,772	149
	Lr-Sr	1B	8.1	9.4	633,768,557	643,147,984	65	5.4	621,535,609	626,964,388	126
	Sr-Yr	5B	9.2	8.5	539,490,416	548,062,568	66	8.5	524,690,853	533,188,305	183
	Lr-Yr	7A	5.2	8.1	63,077,552	71,199,889	31	8.8	66,789,819	75,585,519	124
	Lr-Sr	7B	12.1	18	477,086,379	495,127,476	74	17.3	458,160,404	475,490,074	234
SEEDLING STAGE	Lr-Sr	2A	0	0.002	12,524,633	12,527,343	2	0.003	12,100,642	12,104,031	14
	Lr-Sr-Yr	4A	16.9	15.5	709,687,326	725,242,669	104	25.7	710,004,929	735,763,580	403
	Lr-Sr	6A	0.6	0.001	550,087,569	550,089,292	2	0.2	544,414,000	544,633,493	7
	Lr-Sr	6A	7.9	8.6	609,886,741	618,476,448	83	11.1	601,918,665	613,056,535	289
	Lr-Sr	7A	10.3	0.001	201,007,091	201,008,370	2	18.5	143,882,846	162,397,379	260
	Lr-Sr	1B	4.1	5	658,520,442	663,589,224	32	1.2	649,591,595	650,844,149	25
	Lr-Sr	2B	1.3	5.7	425,532,088	431,218,588	13	5.7	417,050,136	422,729,421	77
	Lr-Sr	2B	7.9	21	591,667,203	612,649,466	98	4.6	601,016,355	605,660,542	81
	Lr-Sr	2B	8	16.8	671,088,315	687,952,296	92	15.4	664,033,146	679,420,589	211
	Lr-Sr	2B	8.6	15.4	763,485,404	778,912,280	107	15.5	752,715,644	768,251,472	195
	Lr-Sr	5B	10.6	37.3	376,620,342	413,965,293	150	17.6	362,192,026	379,816,932	225
	Lr-Yr	5B	9.8	2	693,481,296	695,525,558	21	2.1	682,179,943	684,275,191	62
	Lr-Sr	7B	5.7	3.7	704,100,460	707,804,594	37	6.2	676,095,654	682,337,834	100
	Lr-Yr	7B	5	6.3	724,312,585	730,642,710	25	6.4	696,935,488	703,382,762	115
	Lr-Yr	7B	12.2	11.3	739,046,607	750,372,923	68	7.4	714,375,702	721,753,440	151

**Table 6** reports the physical intervals corresponding to the genetic ones and the number of functional categories annotated in the QTL regions. The physical intervals of QTLs were very similar in both genomes in terms of size, except for some regions of chromosomes 4A, 7A, 2B, and 5B, for which a bigger interval was found on the Svevo genome and *vice versa* (**Table 6**). By contrast, the number of annotated genes in the intervals varied, ranging from two genes on 2A, 6A, and 7A to 150 on 5B in the Zavitan genome, and from seven genes on 6A to 403 on 4A in the Svevo durum wheat genome. In general, a larger number of candidate genes were found in the Svevo genome than in Zavitan, the exceptions being for chromosomes 1B and 2B. Genes annotated in defense-related functional categories were found in all the considered genomic regions of both genomes, except for one region of chromosome 6A (IWB13129), where no defense-related genes were identified either in the Svevo or Zavitan genomes (**Supplementary Materials 8, 9**). In particular, an array of genes encoding the resistance (R-gene) proteins NBS-LRRs (nucleotide-binding site and leucine-rich repeats) and RPM1 (resistance to *Pseudomonas syringae* protein 3), the defense-related proteins RGA2 (Rho-type GTPase-activating protein) and RPP13 (recognition of *Peronospora parasitica* 13), and kinases, receptor-like kinases, PR (pathogenesis-related) proteins, Pm-like proteins, ATP-binding cassette (ABC) transporters, ankyrin repeat family proteins, and sugar transporters were found in the regions corresponding to the resistance QTL acting against two or three species. Additionally, transport receptors, different kinds of transcription factors, and signal transduction pathway proteins were identified, with annotations fully described in **Supplementary Tables 8**, **9**. Moreover, in general, across seedling and adult plant resistance QTLs, not only single annotated genes but also significant clusters of defense-related genes were found at QTLs on chromosomes 4A, 6A, 1B, 2B, and 7B in the Svevo and Zavitan genomes. A gene annotated as *Lr21* in the Svevo genome was identified within the QTL explaining seedling leaf rust and stem rust resistance on chromosome 7B, although at 1.9 MB from the peak marker (**Supplementary Material 8**). Functional annotations of genes corresponding to the peak markers of the QTL regions related to plant disease resistance are also reported in **Tables 4**, **5**. In particular, with regards to APR QTLs (**Table 4**), disease-related annotations were found for two peak QTL markers on chromosome 1B (at 43.5 cM and 115 cM) and for one on 5B that respectively corresponded to a shikimate kinase like 2 protein, an imidazole glycerol phosphate synthase subunit HisF, and a UTP-glucose-1-phospate uridylyltransferase. No functionally annotated gene was found for the peak marker IWB41378 on 1B; however, hits with genes from categories not related to pathogen resistance mechanisms were found for the two peak markers of 7A and 7B (**Table 4**). With respect to MTAs for rust resistance at the seedling stage, all genes corresponding to the peak markers were annotated as disease-related, except those corresponding to the MTAs on 2B (IWB70131), 5B (IWB72712), 6A, and 7A (**Table 5**). The peak marker for the resistance QTL effective against all three rusts on chromosome 4A corresponded to an acetyltransferase gene. Notably, well-known resistance-related genes, such as NBS-LRRs and receptor-like protein kinases, and complex resistance genes, such as Rp1, were shown to correspond to the MTAs IWB66736 on 2A, IWB55526 on 2B, and IWB38104 and IWB9405 on 7B, respectively (**Table 5**). The MTA IWB71824 on chromosome 1B corresponded to a subtilisin-like protein, whereas a chaperone protein dnaJ and a callose synthase corresponded to the hits of MTAs IWB448 and IWA8055 on chromosome 2B, respectively. Moreover, an ADP-ribosylation factor GTPase-activating protein was the candidate for the MTA IWA6468 on 5B. A mediator of RNA polymerase II transcription subunit 15a corresponded to the MTA IWB71982 located on 7B. Finally, the peak MTA of IWB59762 on 2B corresponded to cleavage and polyadenylation specificity factor subunit 5.

## 4 Discussion

Resistance to fungal diseases, in particular to the rusts, is an important target for modern wheat improvement. The most sustainable way to limit yield reductions due to the rusts is to identify new resistance loci in diverse germplasm panels and include them in breeding programs. The reaction of a given genotype to the rust pathogens can vary strongly with respect not only to the species but also to the races within a species and to the environment in which the experiment is carried out. For these reasons, genotypes should be tested for their reaction to the rusts across a range of environments to obtain robust information about their general level of resistance.

In the present investigation, we conducted a genome-wide association study by exploiting a structured panel of tetraploid wheat accessions, comprising of a large set of durum wheat cultivars and a representative sample of other *T. turgidum* evolutionary lineages, including wild and domesticated accessions. Rust resistance phenotypes were investigated across a large number of environments and experimental conditions, i.e., in 28 evaluation tests with three rust species, of which 15 experiments were conducted on adult plants and 13 on seedling plants (**Table 1**). The number of field trials allowed us to assess rust resistance across a wide range of environmental conditions and variable populations of the rust pathogens and compare the field results with those conducted under controlled environments, in which seedlings were challenged with 13 isolates of the three rusts. Resistance sources were identified among domesticated accessions of *T. turgidum* but also within the durum wheat germplasm. Examples are represented by the durum wheat cultivars Altar 84, Granizo, and Grazia, which exhibited a strong resistance phenotype to all races of the stem rust and stripe rust pathogens tested, and by the *T. turgidum* ssp. *polonicum* accession PI 366117, which was found to be resistant to all *Pgt* and *Pst* races and the *Pt* race PSB14. Further studies can be conducted to elucidate the genetic bases of resistance in these highly resistant genotypes. Regardless, Altar 84, Granizo, and Grazia may be useful for developing resistant wheats in breeding programs. The transfer of resistance from the less adapted *T. turgidum* ssp. *polonicum* requires more pre-breeding work to avoid introducing deleterious alleles through linkage drag.

The analysis of LD in the three subsamples of the collection revealed a more rapid decay in the Q2 subgroup than in the durum subsample and the whole collection, which was expected, and in accordance with that observed in a larger collection (nearly 1,800 genotypes) comprising wild and domesticated tetraploid wheat accessions and durum wheat varieties (Maccaferri et al., 2019). The LD values observed for the tetraploid germplasm panel used in this study suggest a good resolution for the GWAS, particularly for the Q2 subsample.

Highly significant QTLs were identified in this genome-wide association study, some of which conferred APR and seedling resistance and explained a high portion of the phenotypic variation. Among the most important QTLs identified, one on chromosome 4A is noteworthy because it confers resistance against all three rust species at the seedling stage, tagged by a peak marker with a very low *P*-value (1.13E-17), and explained up to 30% of the phenotypic variation for resistance to both Spanish *Pt* races (CONDESA and CONIL). Other notable QT loci found included one on chromosome 7A, identified by the peak marker IWB64911, that explained up to 50% of the phenotypic variation for field APR to *Pst*, one on chromosome 7B, tagged by MTA IWB71982, that explained up to 49% of the phenotypic variation for resistance to the *Pst* race PSTv37, and the MTA IWB38104, which explained up to 37% of the variation for stem rust resistance with the race JRCQC. Such loci are of potential interest for future gene cloning and functional characterization or their employment in marker-assisted breeding schemes using KASP or other breeder-friendly marker technologies.

The majority of the QTLs identified in this study are potentially coincident with other QTLs and MTAs reported in the literature for tetraploid wheat (**Table 4**). Yet, two novel QTLs were identified: one on chromosome 5B (Yr-Sr), for adult plant resistance (104 cM), and one on 2B (Lr-Sr), for seedling resistance (182.7 cM). Two seedling resistance QTLs against two rust pathogens (Lr-Sr on 2B at 137.9 cM and Lr-Sr on 6A at 126.7 cM) in this study colocalized with QTLs for resistance to all the three rust pathogens identified in Indian spring wheats evaluated in both controlled and field trials (Kumar et al., 2020). In most cases of map correspondences with previous studies, these regard QTLs involved in the response to the same rust pathogen; for example, the QTL Lr-Yr on 7B (at 187.5 cM), which is coincident with a QTL for Lr resistance (based on SNP IWB71560, Marone et al., 2009) and one for Yr resistance (IWB21278; Liu et al., 2017). However, there are also many cases of QTLs putatively mapping to the same positions in different reports, contributing to the resistance to different rust species, suggesting that the number of loci protecting against different rust pathogens could be higher than previously thought. For example, the region conferring resistance to stripe and leaf rust on 7A at the adult plant stage was coincident with loci previously identified for resistance to stem rust (Sr) under natural infection, based on the markers IWB8374 and IWA8390 (Letta et al., 2013; Aoun et al., 2019). Interestingly, as no cataloged rust resistance genes have been reported in durum or bread wheat on chromosome 7AS, the QTL identified in this study could be novel. Similar results were also found for seedling stage resistance QTLs. The three regions on chromosome 1B, all for Lr-Sr, were coincident with previously identified MTAs for Yr resistance based on the markers IWB72789, IWB59152, and IWB51279 (Liu et al., 2017); these regions might represent loci involved in resistance to all rusts or clusters of genes with different specificities. Moreover, the region on 2B at 95.2 cM (Lr-Sr) was coincident not only with MTAs for leaf and stem rust resistance but also with a locus for Yr resistance previously identified in controlled conditions, based on the marker Xgwm374 (Naz et al., 2012). The region on chromosome 4A found to harbor a QTL for resistance against all three rust species in this study is associated with the presence of QTLs for Yr (Liu et al., 2017) and Sr resistance (Letta et al., 2013; Saccomanno et al., 2018; Saini et al., 2018), but no loci for durum wheat leaf rust resistance were retrieved in the same genomic region in the literature; therefore this common QTL could represent a new multi-pathogen complex.

The positions of QTLs against more than one rust pathogen identified in this work have also been compared with known bread/durum wheat resistance (R) genes (**Figure 1**). None of the known (and cloned) R genes have been found to correspond to the MTAs identified at the adult plant stage, whereas the positions of several identified QTLs for seedling resistance are coincident with known race-specific genes (**Figure 1**). The MTA IWB66736 on 2A could be colocalized with the R gene *Lr17a*, based on the position of close markers Xgwm636 and Xwmc407 (Xue et al., 2018), and *Sr32* (a gene effective against the strain TTKSK), based on the position of the microsatellite Xbarc124 (Yu et al., 2014). Wheat chromosome 2B is very rich in resistance genes. Among them, *Sr36*, linked to Xwmc477 and Xgwm319 (Jin et al., 2009), could be co-mapped with IWB448 (a peak marker of QLrSr(S)-2B.1, **Table 5**); a second R gene, *Sr9h*, can be considered coincident with IWB55526, a peak marker of QLrSr(S)-2B.3 (**Table 5**), because of the position of the marker Xgwm47. Interestingly, in the same region of the QTL identified on 4AL and involved in resistance against all of the rusts, one Sr gene (*SrND463*, based on the marker Xwmc219 and reported to be moderately effective against the *Pgt* races of the Ug99 lineage, including TKTTF) and two *Yr* genes—*Yr51* from the map in Randhawa et al., 2014, and *Yr60*, also associated with the marker Xwmc219 (MAS Wheat website, ucdavis.edu)—have been described previously. No described *Lr* genes are present in this region, suggesting that the QTL common to the three rusts could have identified a new multigenic complex. However, the question of whether the conferring of resistance by QTLs to multiple rust species is due to tightly linked/clustered paralogs or to single genes with pleiotropic effects cannot be resolved from this study and should be investigated in the future.

Within all the genomic regions detected, we identified several candidate genes known to have a *bona fide* role in resistance, such as NBS-LRR genes, protein kinases, or complex resistance genes (Rp). In particular, four QTL peak markers were directly annotated as NBS-LRR genes, protein kinases, and Rp1-like proteins. Interestingly, among them, some were positioned in the same regions where resistance genes for rusts have previously been identified in wheat. An example is the QTL for Lr-Yr resistance on 7B (211.5 cM), the peak marker of which, IWB9405, was found to correspond to a Rp1-like protein in the Svevo genome, in turn coincident not only with a rust-specific gene (*Lr14a*; Herrera-Fossel et al., 2008) but also the APR gene *Lr68* (Herrera-Fossel et al., 2012). These could represent two closely linked genes, but allelism tests should be performed to determine the true relationships between the detected loci and previously reported genes. Other functional categories were annotated in the QTL regions, together with well-known disease-related classes, such as callose synthase, which plays a role in pathogen-induced callose deposition at sites of infection (Ellinger and Voigt, 2014), or sugar transporters, which could be responsible for resistance phenotypes, e.g., the cloned gene *Lr67* (Moore et al., 2015).

The QTL controlling the response against the three different rust pathogens on chromosome 4A has a marker peak corresponding to an acetyltransferase, a component of the pyruvate dehydrogenase complex. Although a direct linkage to a resistance mechanism is not evident, Balmant et al. (2015) demonstrated that a redox-responsive protein was involved at the early stages of *Pseudomonas syringae* infection in tomato. Mediator of RNA polymerase II transcription subunit 15a, a candidate gene for the MTA IWB71982 on 7B (**Table 5**), was demonstrated to be involved in plant defense in tobacco (Lee et al., 2003). Finally, five clusters of defense-related genes were identified in the QTL regions on chromosomes 4A, 6A, 1B, 2B, and 7B, confirming previous results reported in wheat, i.e., that QTLs for resistance to fungal diseases like rusts often coincide with clusters of defense-related genes. Although this is not surprising given that NBS-LRR genes can occur in clusters (Chen et al., 2015), it can be hypothesized that one or more elements composing such clusters could be the causative genetic variants at the basis of the QTL phenotypic effects.

Minor effect QTLs that alone are not significant for effective resistance in the crop could interact with other QTLs and positively increase the resistance, as demonstrated by Singh et al. (2013). Therefore, the array of minor-effect QTLs identified in this study could also enhance disease resistance if used in combination with main-effect QTLs. With this in mind, the genotypic and phenotypic datasets generated in this study deserve to be exploited by models of genomic selection.

## 5 Conclusions

This study reports valuable genetic variation for resistance to multiple rust pathogens in seedling tests and field trials in a tetraploid wheat panel. Some durum wheat varieties and one *T. polonicum* (PI 366117) genotype were highly resistant in multiple environments and against multiple rust species. The GWAS revealed a highly significant QTL for resistance to all three rust pathogens of wheat. Six QTLs for APR to two different rust pathogens were identified. Additionally, 15 QTLs for seedling resistance to two rust species were found, with most being effective against leaf rust and stem rust. One QTL for seedling resistance on chromosome 4A was the only one conferring resistance to all three rust pathogens. The identified QTLs with the highest explained phenotypic variances are suitable targets for cloning and functional characterization. For breeding uses, the identified QTL with major effects on the resistance are available for pyramiding and marker-assisted selection, by the development of KASP markers useful to screen germplasm panels; while the whole set of QTL, for genomic selection of rust resistance in wheat.

## Data availability statement

The data presented in the study are deposited in the FigShare repository, accession number 24183198 (https://figshare.com/articles/dataset/CRdataset/24183198).

## Author contributions

DM: Writing – original draft, Writing – review & editing, Formal analysis, Investigation, Validation. GL: Formal analysis, Writing – original draft, Writing – review & editing, Data curation, Methodology, Supervision. AS: Data curation, Formal analysis, Methodology, Writing – review & editing, Investigation, Resources. GP: Data curation, Formal analysis, Writing – review & editing, Software, Validation, Visualization. CG: Data curation, Formal analysis, Validation, Writing – review & editing, Methodology. PD: Writing – review & editing, Conceptualization. AM: Conceptualization, Writing – review & editing, Validation, Visualization. AG: Validation, Visualization, Writing – review & editing. KA: Validation, Visualization, Writing – review & editing, Data curation, Investigation, Methodology. FB: Data curation, Investigation, Methodology, Validation, Visualization, Writing – review & editing. MW: Data curation, Investigation, Methodology, Writing – review & editing. XC: Data curation, Methodology, Writing – review & editing, Supervision. DR: Data curation, Methodology, Writing – review & editing, Formal analysis, Investigation, Validation. OM: Data curation, Formal analysis, Investigation, Methodology, Writing – review & editing. BS: Data curation, Formal analysis, Methodology, Writing – review & editing, Supervision. NP: Methodology, Supervision, Writing – review & editing, Conceptualization, Funding acquisition, Writing – original draft.
