# Supplementary material for: Genome-wide association study of common resistance to rust species in tetraploid wheat
Source: Front Plant Sci. 2024 Jan 3;14:1290643. doi: 10.3389/fpls.2023.1290643 (PMC10792004; doi:10.3389/fpls.2023.1290643)

**SM 1 - T1.** Statistical parameters of reactions of the tetraploid wheat collection to stem, stripe and leaf rust at the adult plant stage, in field trials. The trial code reports the institution, the first letter of the environment, the first letter of the sowing time and the initial letter of the rust species in upper case (S, stem; L, leaf; Y, stripe rust). CV: coefficient of variation; *H_2_*: broad sense heritability; LSD: least significant differences; RCD: replicated check design; RBD: random block design.

| **Species** | **Institution (Environment - Sowing time)** | **Trial Code** | **Model** | **Mean** | **Min-Max** | **CV (%)** | ***H_2_*** | **LSD** |
| --- | --- | --- | --- | --- | --- | --- | --- | --- |
| **Stem Rust** | University of Minnesota (Saint Paul - Spring 2015) | UMNssS | RCD | 20.0 | 0-90 | 26.8 | 91.5 | 13.6 |
| **Stripe Rust** | USDA (Pullman - Spring 2015) | USDApsY | RCD | 13.6 | 0-100 | 40.5 | 77.2 | 37.6 |
|  | USDA (Pullman - Autumn 2015) | USDApaY | RCD | 26.0 | 0-100 | 27.3 | 77.3 | 45.8 |
|  | USDA (Mt. Vernon - Spring 2015) | USDAvsY | RCD | 33.7 | 0-100 | 33.4 | 74.1 | 71.4 |
|  | ICARDA (Marchouch - Autumn 2015) | ICARDAmaY | RCD | 34.7 | 0-100 | 27.6 | 82.3 | 61.1 |
|  | CREA (Foggia - Winter 2015) | CREAfw15Y | RBD | 31.4 | 0-90 | 40.4 | 65.4 | 34.6 |
|  | CREA (Foggia - Winter 2016) | CREAfw16Y | RCD | 27.5 | 0-80 | 29.0 | 74.6 | 60.5 |
|  | CIMMYT (Toluca - Summer 2016) | CIMMYTtsY | RBD | 46.9 | 0-100 | 15.6 | 90.1 | 22.4 |
|  | CIMMYT (El Batan - Summer 2016) | CIMMYTbsY | RBD | 7.8 | 0-60 | 49.9 | 62.0 | 12.1 |
| **Leaf Rust** | ICARDA (Marchouch - Autumn 2015) | ICARDAmaL | RCD | 0.6 | 0-20 | 36.7 | - | 13.1 |
|  | ICARDA (Allal Tazi - Autumn 2016) | ICARDAaaL | RCD | 35.5 | 0-90 | 20.3 | 93.1 | 18.5 |
|  | CREA (Foggia - Winter 2015) | CREAfwL | RBD | 24.1 | 0-90 | 41.8 | 75.8 | 31.8 |
|  | CSIC (Cordoba - Autumn 2015) | CSICcaL | RBD | 14.3 | 0-80 | 43.5 | 78.5 | 15.4 |
|  | CIMMYT (El Batan - Summer 2016) | CIMMYTbsL | RBD | 31.5 | 0-90 | 22.7 | 93.6 | 17.0 |
|  | CIMMYT (Obregon - Autumn 2016) | CIMMYToaL | RBD | 40.0 | 0-100 | 28.4 | 86.5 | 28.3 |

**SM 1 - T2.** Statistical parameters of reactions of the tetraploid wheat collection to stem, stripe and leaf rust, at the seedling stage in controlled environments. The trial code corresponds to the rust race used for phenotyping. CV: coefficient of variation; *H_2_*: broad sense heritability; LSD: least significant difference.

| **Species** | **Institution (Race)** | **Trial Code** | **Mean** | **Min-Max** | **CV (%)** | ***H_2_*** | **LSD** |
| --- | --- | --- | --- | --- | --- | --- | --- |
| **Stem Rust** | University of Minnesota (TTTTF) | TTTTF | 6.6 | 0-10 | 9.5 | 98.1 | 1.3 |
|  | University of Minnesota (TPMKC) | TPMKC | 6.7 | 0-10 | 10.5 | 96.8 | 1.4 |
|  | University of Minnesota (TRTTF) | TRTTF | 6.4 | 0-10 | 11.5 | 96.4 | 1.5 |
|  | University of Minnesota (JRCQC) | JRCQC | 7.8 | 0-10 | 9.7 | 91.2 | 1.5 |
|  | University of Minnesota (TKTTF) | TKTTF | 5.6 | 0-10 | 14.3 | 97.2 | 2.4 |
| **Stripe Rust** | University of Minnesota (PSTV 14) | PSTV14 | 4.0 | 0-8 | 15.4 | 92.7 | 1.2 |
|  | University of Minnesota (PSTV 37) | PSTV37 | 5.2 | 0-9 | 7.74 | 98.4 | 0.8 |
|  | University of Minnesota (PSTV 40) | PSTV40 | 5.2 | 0-9 | 8.43 | 97.7 | 0.9 |
| **Leaf Rust** | University of Minnesota (PSB 14) | PSB14 | 6.7 | 0-9 | 16.1 | 88.8 | 2.1 |
|  | University of Minnesota (SPAIN 52) | SPAIN52 | 7.6 | 0-9 | 17.3 | 77.4 | 2.6 |
|  | CSIC (Villamanrique de la Condesa) | CONDESA | 7.4 | 0-9 | 7.4 | 95.6 | 1.1 |
|  | CSIC (CONIL) | CONIL | 7.6 | 0-9 | 5.8 | 94.8 | 0.9 |

**SM 1 - T3.** ANOVA results - (General linear mixed model) of reactions of the tetraploid wheat collection to stem, stripe and leaf rust at the adult plant stage, in experimental field trials: a) Replicated check design; b) Random Block Design. DF: degrees of freedom; SS: sum of squares; MS: mean square; F: F-ratio; P: probability.

| **a)** |  | **Source** | **DF** | **SS** | **MS** | **F** | **P(>F)** | |
| --- | --- | --- | --- | --- | --- | --- | --- | --- |
| **Stem Rust** | **UMNssS** | Genotype | 204 | 74,601.98 | 365.70 | 11.81 | 0.00 | ** |
|  |  | Residual | 6 | 185.71 | 30.95 |  |  |  |
| **Stripe Rust** | **USDApsY** | Genotype | 228 | 39,306.8 | 172.4 | 4.39 | 0.033 | * |
|  |  | Residual | 6 | 235.7 | 39.3 |  |  |  |
|  | **USDApaY** | Genotype | 221 | 56,911.7 | 257.5 | 4.41 | 0.032 | * |
|  |  | Residual | 6 | 350.0 | 58.3 |  |  |  |
|  | **USDAvsY** | Genotype | 140 | 76,677.8 | 547.7 | 3.87 | 0.045 | * |
|  |  | Residual | 6 | 850.0 | 141.7 |  |  |  |
|  | **ICARDAmaY** | Genotype | 192 | 112,062.3 | 583.7 | 5.64 | 0.017 | * |
|  |  | Residual | 6 | 621.4 | 103.6 |  |  |  |
|  | **CREAfw16Y** | Genotype | 229 | 61,052.28 | 266.6 | 3.93 | 0.01 | * |
|  |  | Residual | 9 | 610.0 | 67.78 |  |  |  |
| **Leaf Rust** | **ICARDAmaL** | Genotype | 190 | 959.96 | 5.05 | 0.18 | 1.00 | ns |
|  |  | Residual | 6 | 171.43 | 28.57 |  |  |  |
|  | **ICARDAaaL** | Genotype | 220 | 181,757.19 | 826.17 | 14.46 | 0.00 | ** |
|  |  | Residual | 6 | 342.86 | 57.14 |  |  |  |

| **b)** |  | **Source** | **DF** | **SS** | **MS** | **F** | **P(>F)** | |
| --- | --- | --- | --- | --- | --- | --- | --- | --- |
| **Stripe Rust** | **CREAfw15Y** | Genotype | 229 | 2,563.17 | 11.19 | 2.89 | 0.00 | *** |
|  |  | Block | 1 | 70.89 | 70.89 | 18.29 | 0.00 | *** |
|  |  | Residuals | 229 | 887.48 | 3.88 |  |  |  |
|  | **CIMMYTtsY** | Genotype | 226 | 2,271.08 | 10.05 | 10.05 | 0.00 | *** |
|  |  | Block | 1 | 4.36 | 4.36 | 4.36 | 0.04 | * |
|  |  | Residuals | 221 | 220.92 | 1.00 |  |  |  |
|  | **CIMMYTbsY** | Genotype | 220 | 883.12 | 4.01 | 2.63 | 0.00 | *** |
|  |  | Block | 1 | 1.49 | 1.49 | 0.98 | 0.32 | ns |
|  |  | Residuals | 210 | 319.98 | 1.52 |  |  |  |
| **Leaf Rust** | **CREAfw15L** | Genotype | 229 | 2,754.87 | 12.03 | 4.14 | 0.00 | *** |
|  |  | Block | 1 | 37.17 | 37.17 | 12.78 | 0.00 | *** |
|  |  | Residuals | 229 | 666.11 | 2.91 |  |  |  |
|  | **CSICcaL** | Genotype | 227 | 1,859.05 | 8.19 | 4.66 | 0.00 | *** |
|  |  | Block | 1 | 0.24 | 0.24 | 0.14 | 0.71 | ns |
|  |  | Residuals | 222 | 390.48 | 1.76 |  |  |  |
|  | **CIMMYTbsL** | Genotype | 220 | 3,882.10 | 17.65 | 15.63 | 0.00 | *** |
|  |  | Block | 1 | 0.82 | 0.82 | 0.73 | 0.39 | ns |
|  |  | Residuals | 210 | 237.17 | 1.13 |  |  |  |
|  | **CIMMYToaL** | Genotype | 224 | 3,977.76 | 17.76 | 7.39 | 0.00 | *** |
|  |  | Block | 1 | 0.57 | 0.57 | 0.24 | 0.63 | ns |
|  |  | Residuals | 218 | 523.80 | 2.40 |  |  |  |

**SM 1 - T4.** ANOVA results - (General linear mixed model) of reactions of the tetraploid wheat collection to stem, stripe and leaf rust at the seedling stage, in controlled environment experiments. DF: degrees of freedom; SS: sum of squares; MS: mean square; F: F-ratio; P: probability.

|  |  | **Source** | **DF** | **SS** | **MS** | **F** | **P(>F)** | |
| --- | --- | --- | --- | --- | --- | --- | --- | --- |
| **Stem Rust** | **TTTTF** | Genotype | 222 | 4,617.8 | 20.8009 | 51.296 | < 2e-16 | *** |
|  |  | Block | 1 | 1.7 | 1.708 | 4.212 | 0.04131 | * |
|  |  | Residuals | 222 | 90 | 0.4055 |  |  |  |
|  | **TPMKC** | Genotype | 224 | 3,500.7 | 15.6282 | 31.2916 | < 2.2e-16 | *** |
|  |  | Block | 1 | 3.9 | 3.9014 | 7.8115 | 0.005642 | ** |
|  |  | Residuals | 224 | 111.9 | 0.4994 |  |  |  |
|  | **TRTTF** | Genotype | 221 | 3,494.4 | 15.8118 | 27.886 | < 2.2e-16 | *** |
|  |  | Block | 1 | 8.4 | 8.3806 | 14.78 | 0.000158 | *** |
|  |  | Residuals | 221 | 125.3 | 0.567 |  |  |  |
|  | **JRCQC** | Genotype | 225 | 1,475.24 | 6.5566 | 11.3985 | <2e-16 | *** |
|  |  | Block | 1 | 1.06 | 1.0611 | 1.8447 | 0.1758 |  |
|  |  | Residuals | 225 | 129.42 | 0.5752 |  |  |  |
|  | **TKTTF** | Genotype | 221 | 5217.5 | 23.6088 | 35.4255 | <2e-16 | *** |
|  |  | Block | 1 | 0 | 0.0027 | 0.0041 | 0.9491 |  |
|  |  | Residuals | 221 | 147.3 | 0.6664 |  |  |  |
| **Stripe Rust** | **PSTV14** | Genotype | 225 | 1,149.45 | 5.1087 | 13.7046 | <2e-16 | *** |
|  |  | Block | 1 | 0.89 | 0.8938 | 2.3978 | 0.1229 |  |
|  |  | Residuals | 225 | 83.87 | 0.3728 |  |  |  |
|  | **PSTV37** | Genotype | 221 | 2,196.35 | 9.9383 | 62.2453 | < 2e-16 | *** |
|  |  | Block | 1 | 0.44 | 0.4383 | 2.7451 | 0.09897 |  |
|  |  | Residuals | 221 | 35.29 | 0.1597 |  |  |  |
|  | **PSTV40** | Genotype | 225 | 1,898.46 | 8.4376 | 43.9114 | <2e-16 | *** |
|  |  | Block | 1 | 0.16 | 0.1561 | 0.8124 | 0.3684 |  |
|  |  | Residuals | 225 | 43.23 | 0.1922 |  |  |  |
| **Leaf Rust** | **PSB14** | Genotype | 225 | 2,318.74 | 10.3055 | 8.8973 | < 2e-16 | *** |
|  |  | Block | 1 | 8.23 | 8.2256 | 7.1016 | 0.00827 | ** |
|  |  | Residuals | 221 | 255.98 | 1.1583 |  |  |  |
|  | **SPAIN52** | Genotype | 223 | 1,741.48 | 7.8093 | 4.4231 | <2e-16 | *** |
|  |  | Block | 1 | 3.01 | 3.0085 | 1.704 | 0.1931 |  |
|  |  | Residuals | 219 | 386.66 | 1.7656 |  |  |  |
|  | **CONDESA** | Genotype | 227 | 1,550.79 | 6.8317 | 22.6045 | < 2.2e-16 | *** |
|  |  | Block | 2 | 3.13 | 1.5637 | 5.1738 | 0.006018 | ** |
|  |  | Residuals | 434 | 131.17 | 0.3022 |  |  |  |
|  | **CONIL** | Genotype | 225 | 867.21 | 3.8543 | 19.3911 | <2e-16 | *** |
|  |  | Block | 2 | 0.46 | 0.231 | 1.1623 | 0.3137 |  |
|  |  | Residuals | 433 | 86.07 | 0.1988 |  |  |  |

**SM 1 - Fig1.** Distribution frequencies of the reactions (disease severity, DS) to stem rust at adult plant stage in the field trials: **a)** Distribution histograms; **b)** Boxplot.


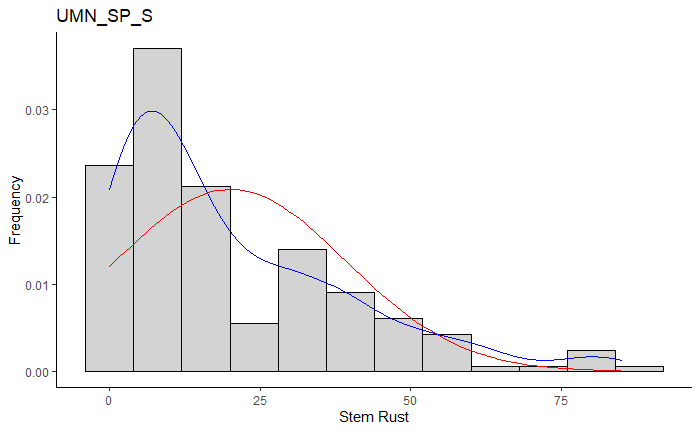


Stem Rust DS

**UMNssS**

**a)**

**b)**

**UMNssS**

**
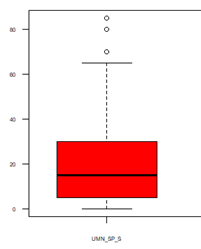
**

**DS**

**SM 1 - Fig2.** Distribution frequencies of the reactions (disease severity, DS) to stripe rust at adult plant stage in the field trials: **a)** Distribution histograms; **b)** Boxplots.

**a)**


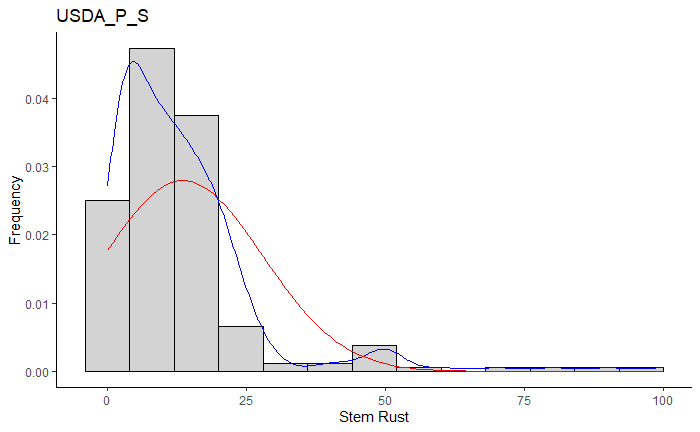


Stripe Rust DS

**USDApsY**


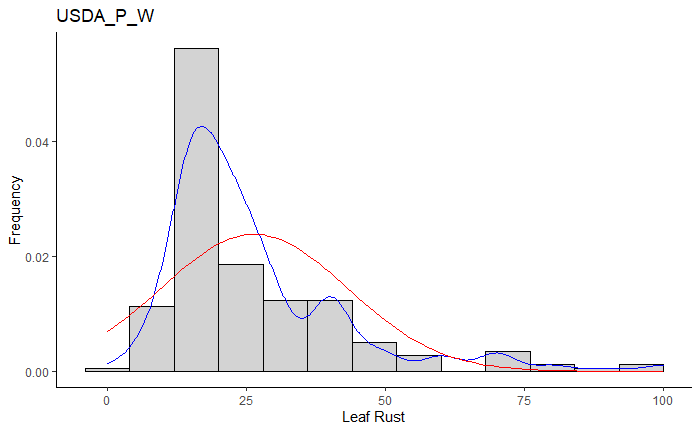


Stripe Rust DS

**USDApwY**


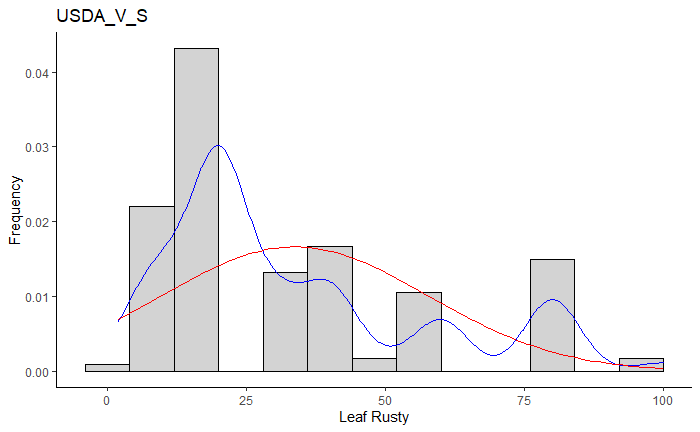


Stripe Rust DS

**USDAvsY**


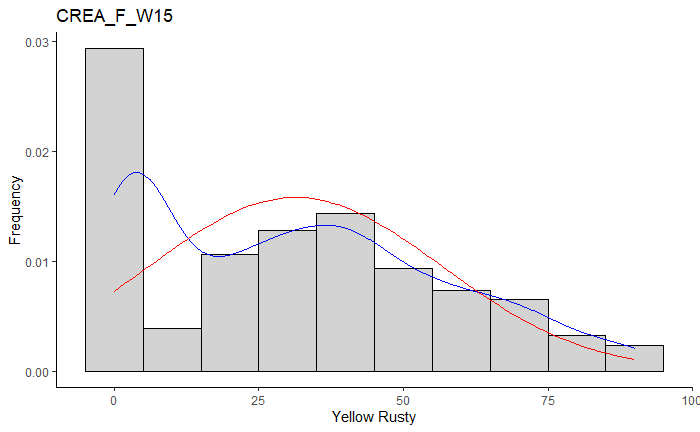


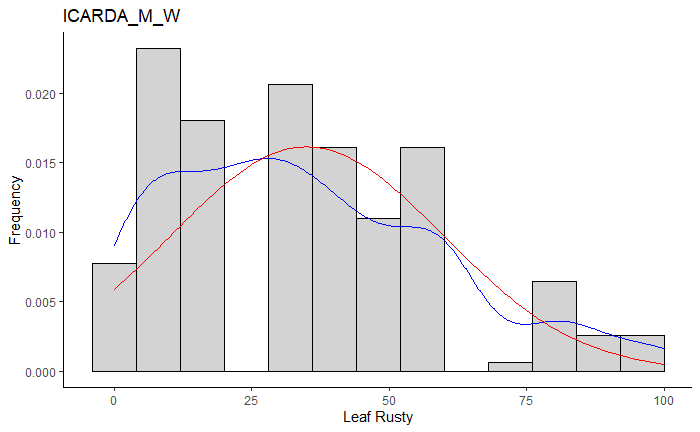


**ICARDAmaY**

Stripe Rust DS

**CREAfw15Y**

**CREAfw16Y**


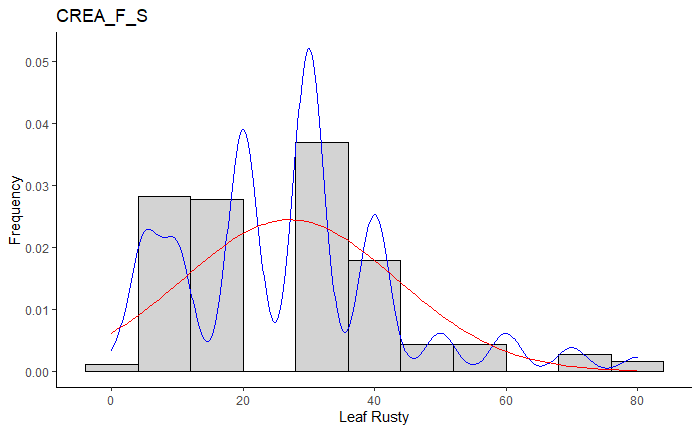


Stripe Rust DS

Stripe Rust DS


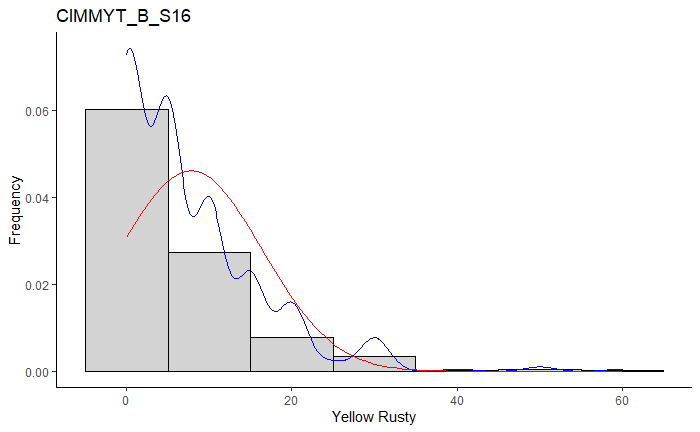


Stripe Rust DS

**CIMMYTbsY**


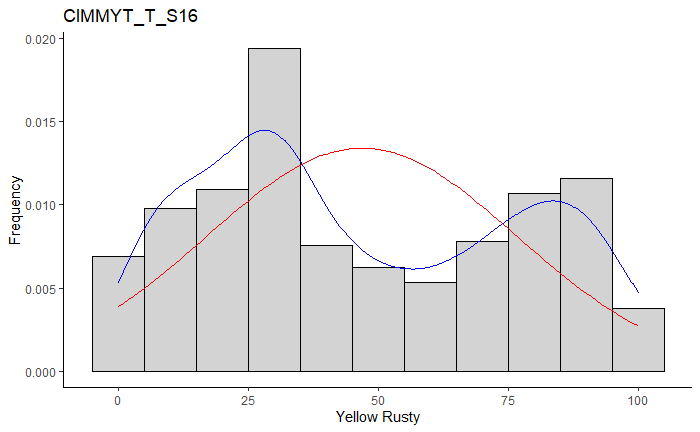


Stripe Rust DS

**CIMMYTtsY**


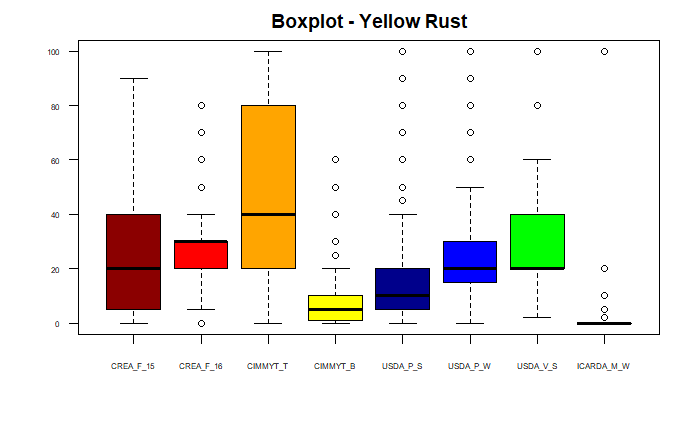


**CREAfw15Y**

**CREAfw16Y**

**CIMMYTtsY**

**CIMMYTbsY**

**USDApsY**

**USDApwY**

**USDAvsY**

**ICARDAmaY**

**b)**

**DS**

**SM 1 - Fig3.** Distribution frequencies of the reactions (disease severity, DS) to leaf rust at adult plant stage in the field trials: **a)** Distribution histograms; **b)** Boxplots.

**a)**


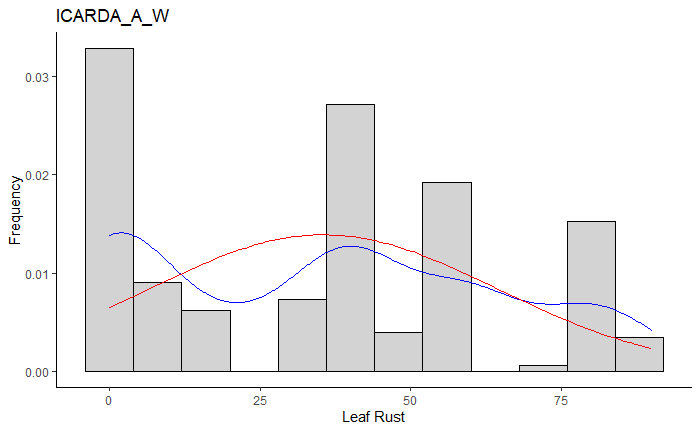

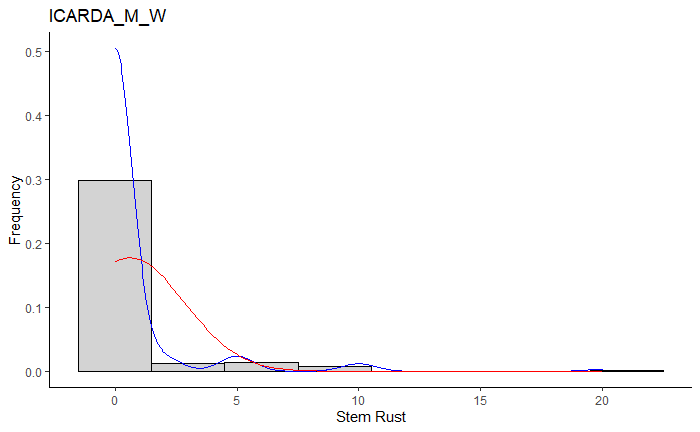


**ICARDAaaL**

**ICARDAmaL**

Leaf Rust DS

Leaf Rust DS


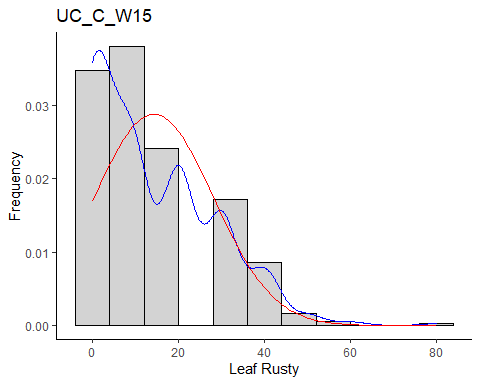


Leaf Rust DS

**CSICcwL**


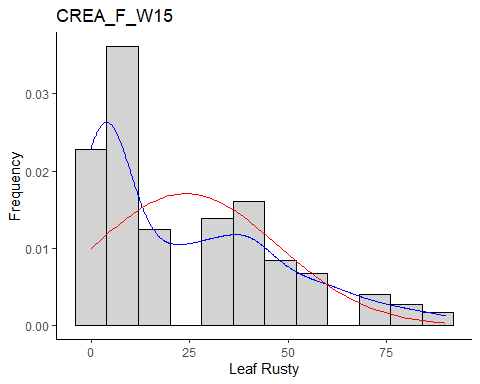


Leaf Rust DS

**CREAfw15L**


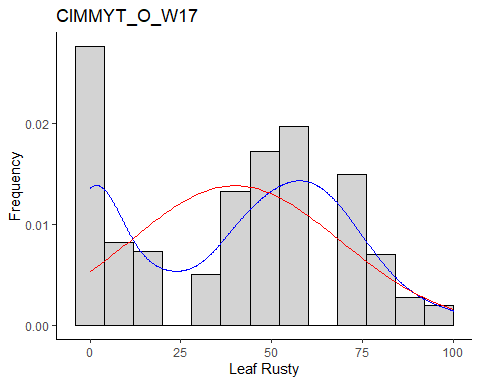


Leaf Rust DS

**CIMMYToaL**


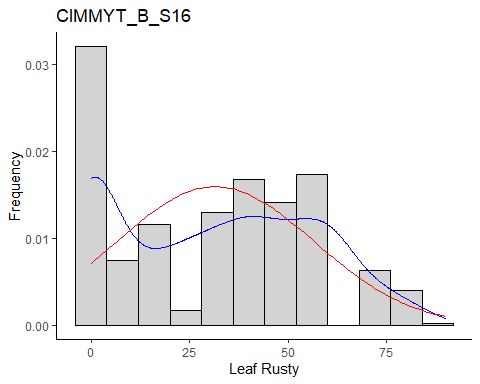


Leaf Rust DS

**CIMMYTbsL**


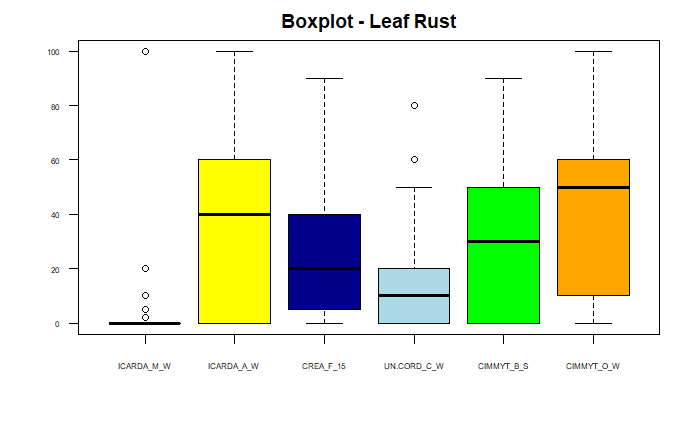


**b)**

**DS**

**CIMMYToaL**

**CIMMYTbsL**

**CSICcwL**

**CREAfw15L**

**ICARDAmaL**

**ICARDAaaL**

**SM 1 - Fig4.** Distribution frequencies of the reactions (infection type, IT) to stem rust at the seedling stage, in controlled environments: **a)** Distribution histograms; **b)** Boxplots.

**a)**

**
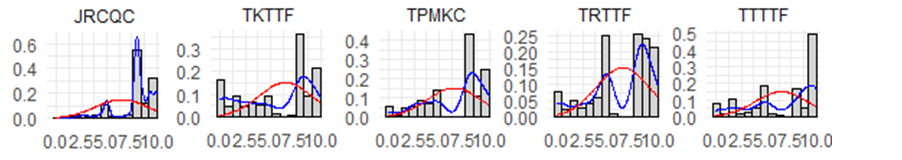
**

**
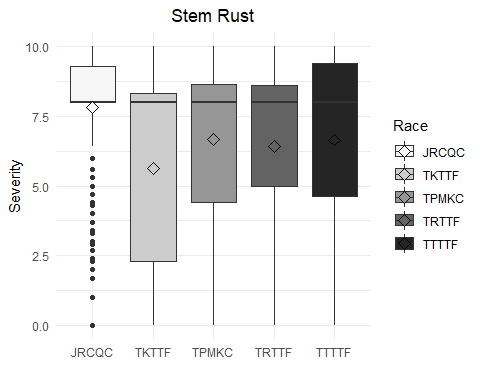
**

**b)**

**IT**

**SM 1 - Fig5.** Distribution frequencies of the reactions (infection type, IT) to stripe rust at the seedling stage in controlled environment: **a)** Distribution histograms; **b)** Boxplots.


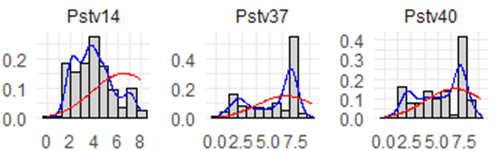


**a)**


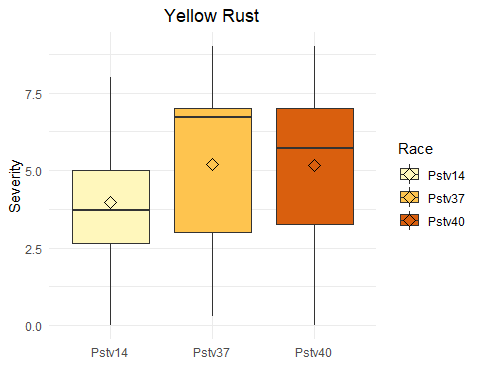


**b)**

**IT**

**SM 1 - Fig6.** Distribution frequencies of the reactions (infection type, IT) to leaf rust at the seedling stage, in controlled environments: **a)** Distribution histograms; **b)** Boxplots.


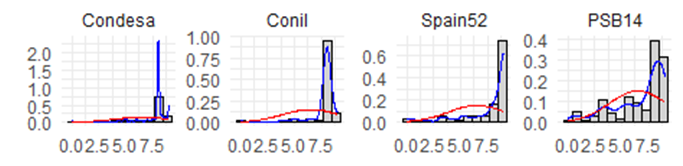


**a)**


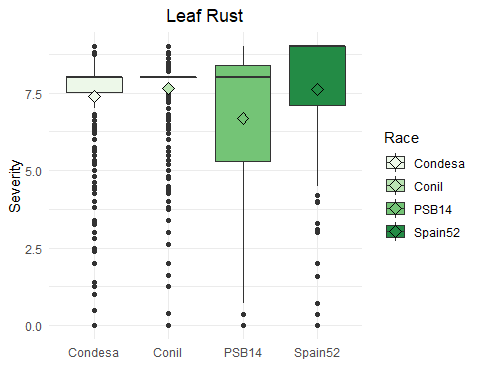


**b)**

**IT**

**SM 1 - Fig7.** Categories of reactions to rusts (Susceptible; Moderately susceptible; Moderately tolerant; Tolerant) at seedling stage and their relative distribution in the panel, against: **a)** stem rust, **b)** stripe rust, **c)** leaf rust.


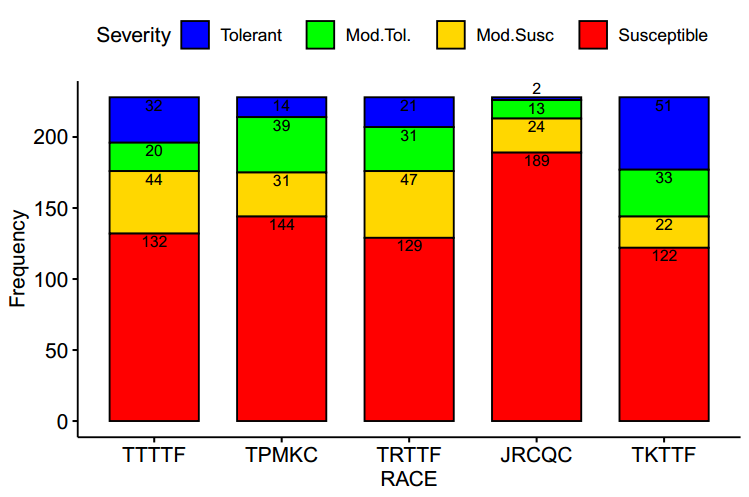


**IT**

**(0-2)**

**(2-4)**

**(4-6)**

**(6-10)**

**a)**


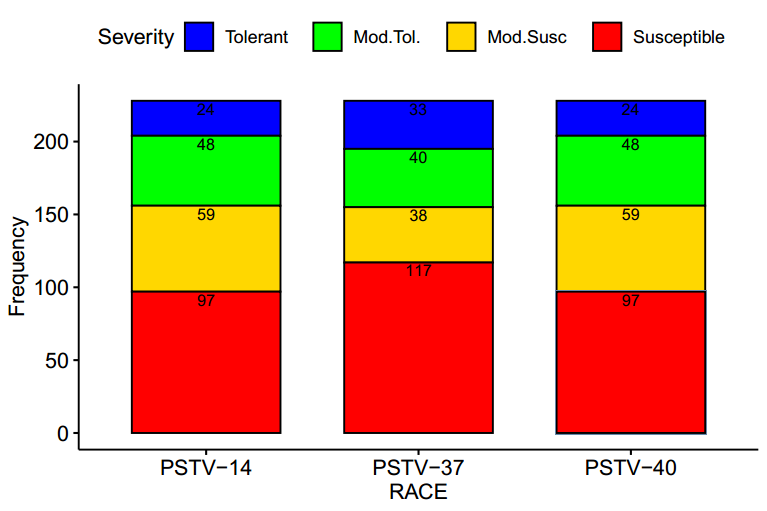


**IT**

**(0-2)**

**(2-4)**

**(4-6)**

**(6-9)**

**b)**


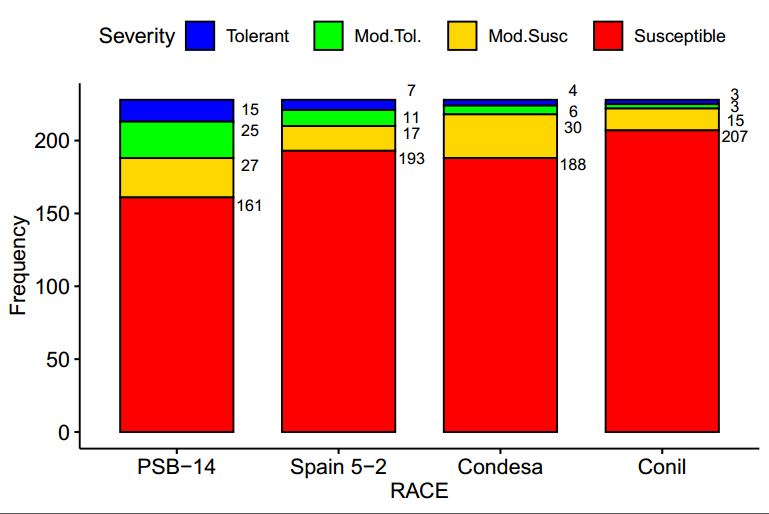


PSTV14

PSTV37

PSTV40

**c)**

**IT**

**(0-2)**

**(2-4)**

**(4-6)**

**(6-9)**

PSB14

SPAIN52

CONDESA

CONIL

RACE

**SM 1 - Fig8.** Phenotypic variation of the reactions of the different tetraploid *Triticum* subspecies to five isolates of stem rust at the seedling stage.

**
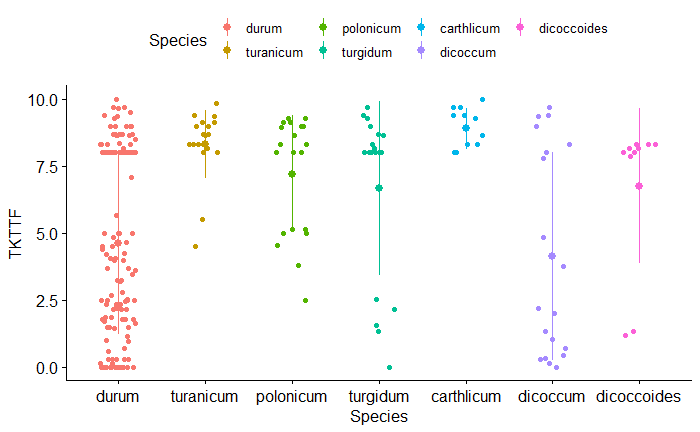

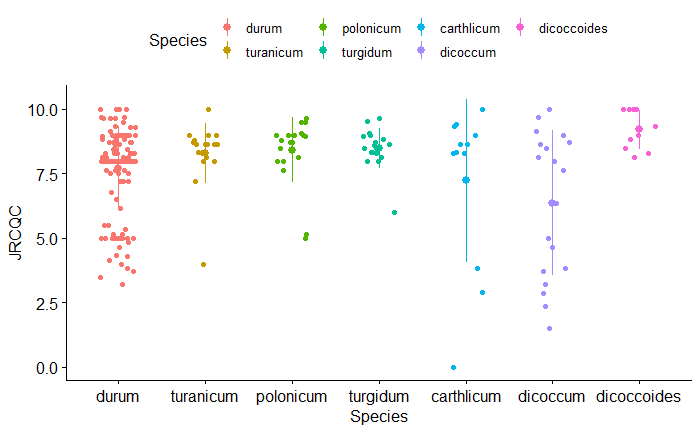
**
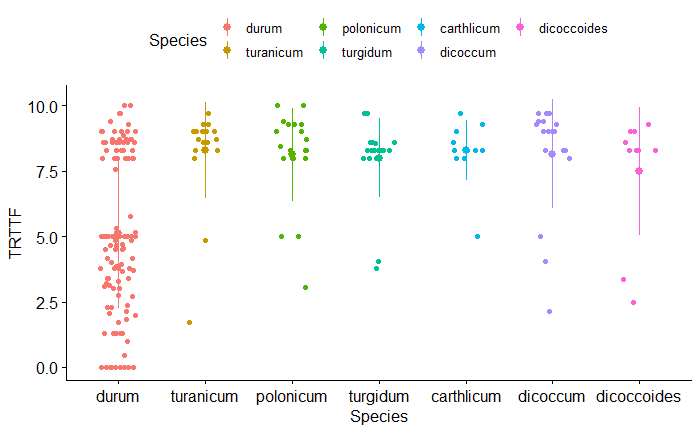

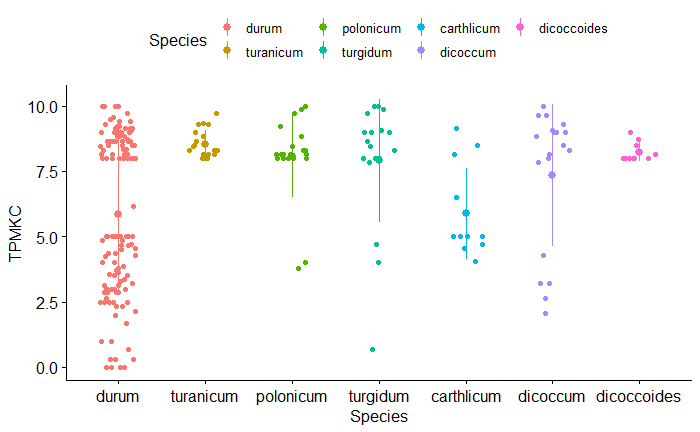

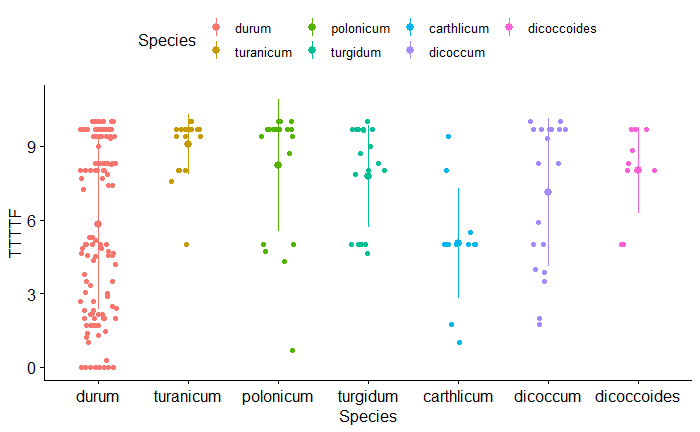


**SM 1 - Fig9.** Phenotypic variation of the reactions of the different tetraploid *Triticum* subspecies to three isolates of stripe rust at the seedling stage.


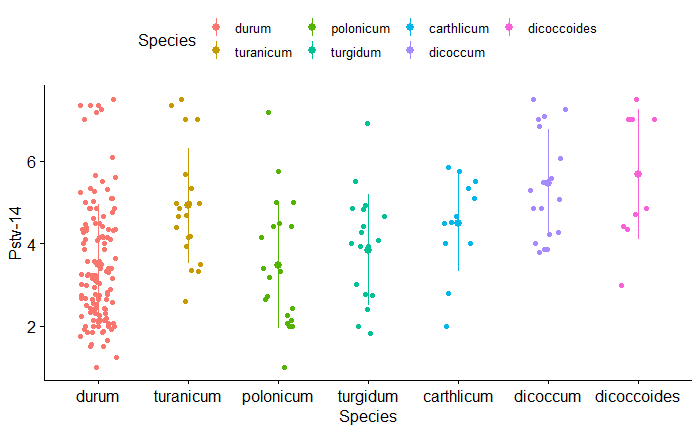

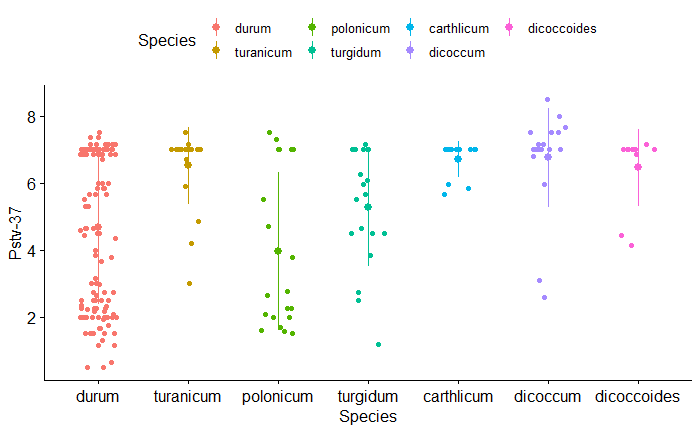


PSTV37

PSTV14


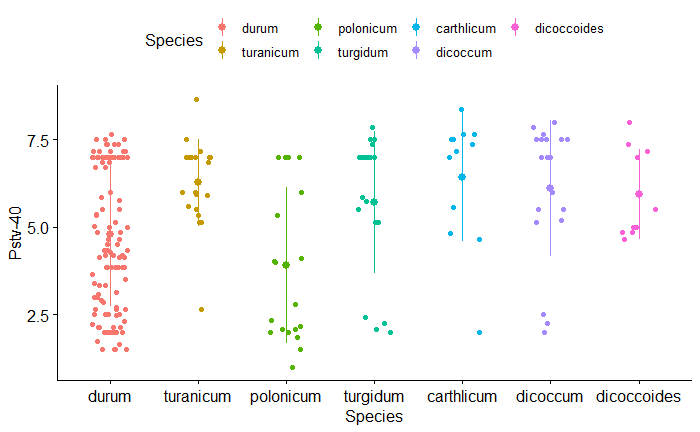


PSTV40

**SM 1 - Fig10.** Phenotypic variation of the reactions of the different tetraploid *Triticum* subspecies to four isolates of leaf rust at the seedling stage.


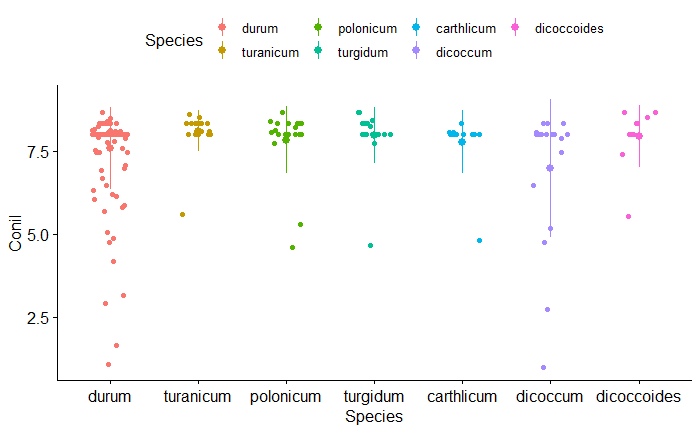

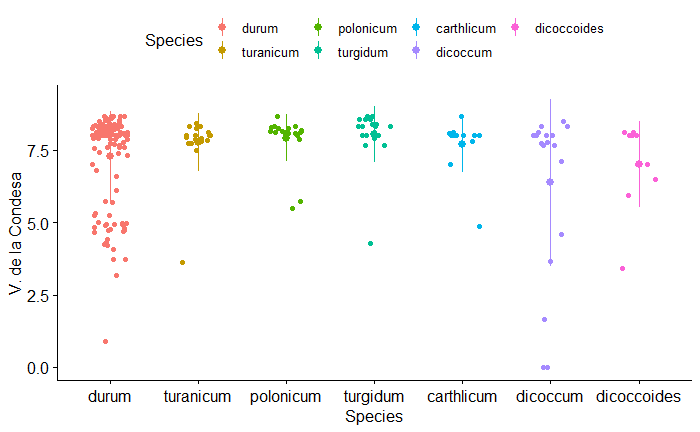


CONIL

CONDESA

**
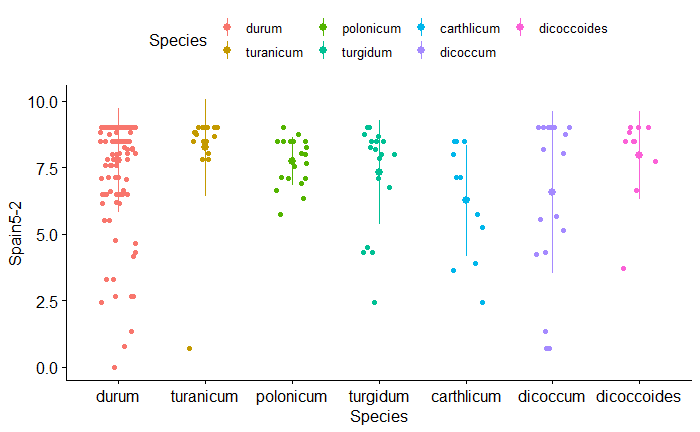

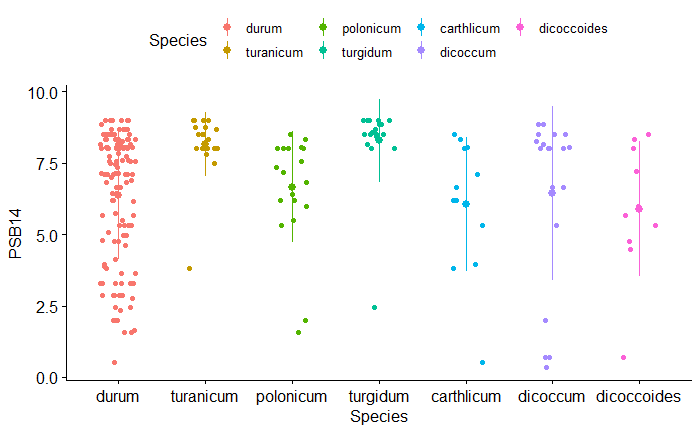
**

SPAIN52

PSB14

**
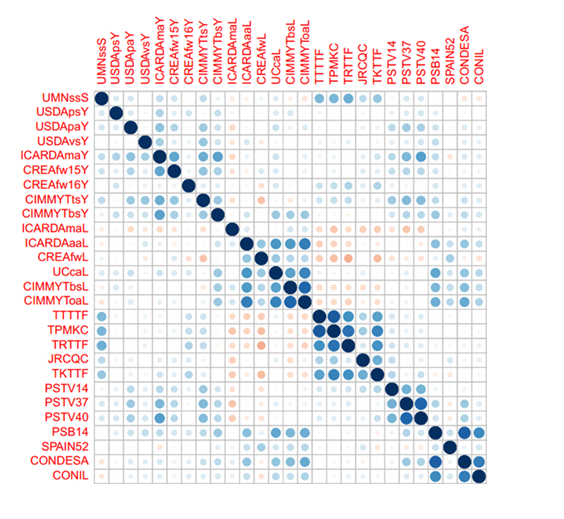
SM 1 - Fig11.** Pearson correlations between all experiments at adult and seedling stage.


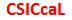


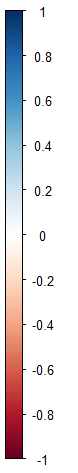


Sr

Yr

Field Trials

Lr


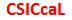


Sr

Controlled Environments

Yr

Lr

**SM 1 - Fig12.** PCA Biplot of stem rust seedling and adult experiments.

**
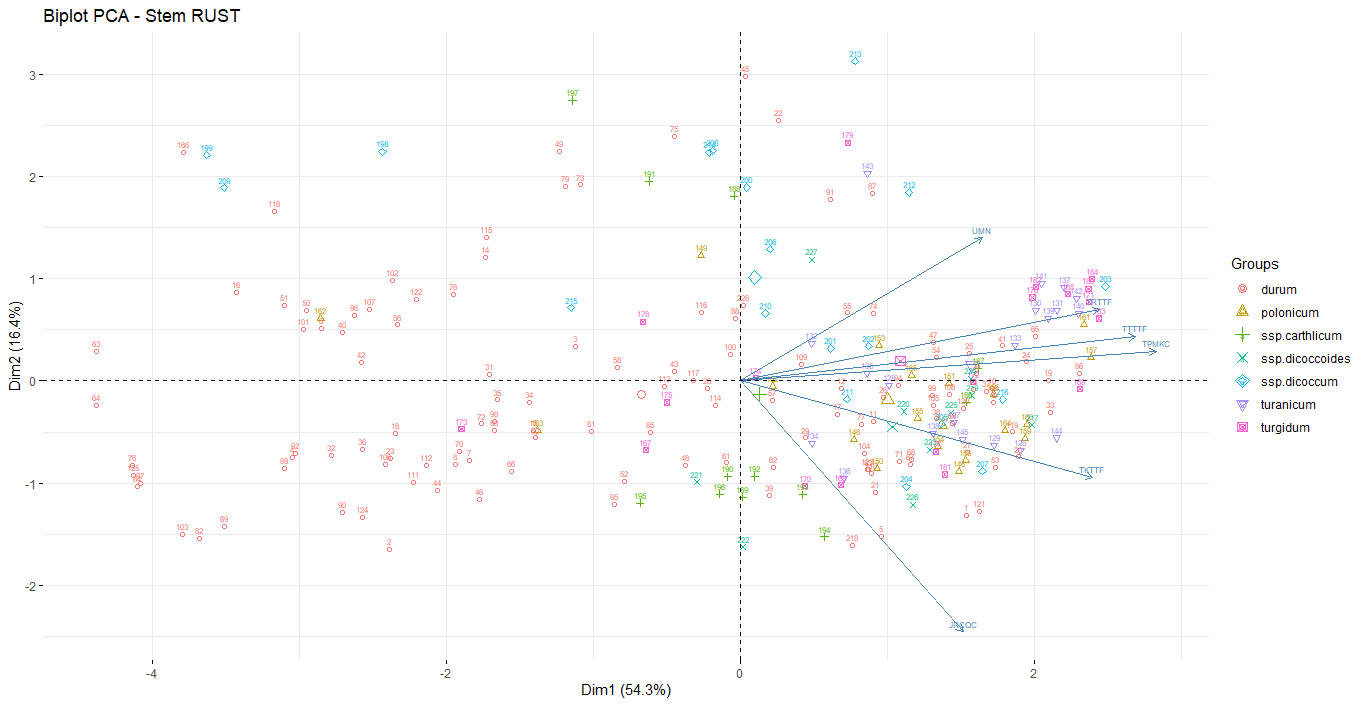
**

**SM 1 - Fig13.** PCA Biplot of stripe rust seedling and adult experiments.

**
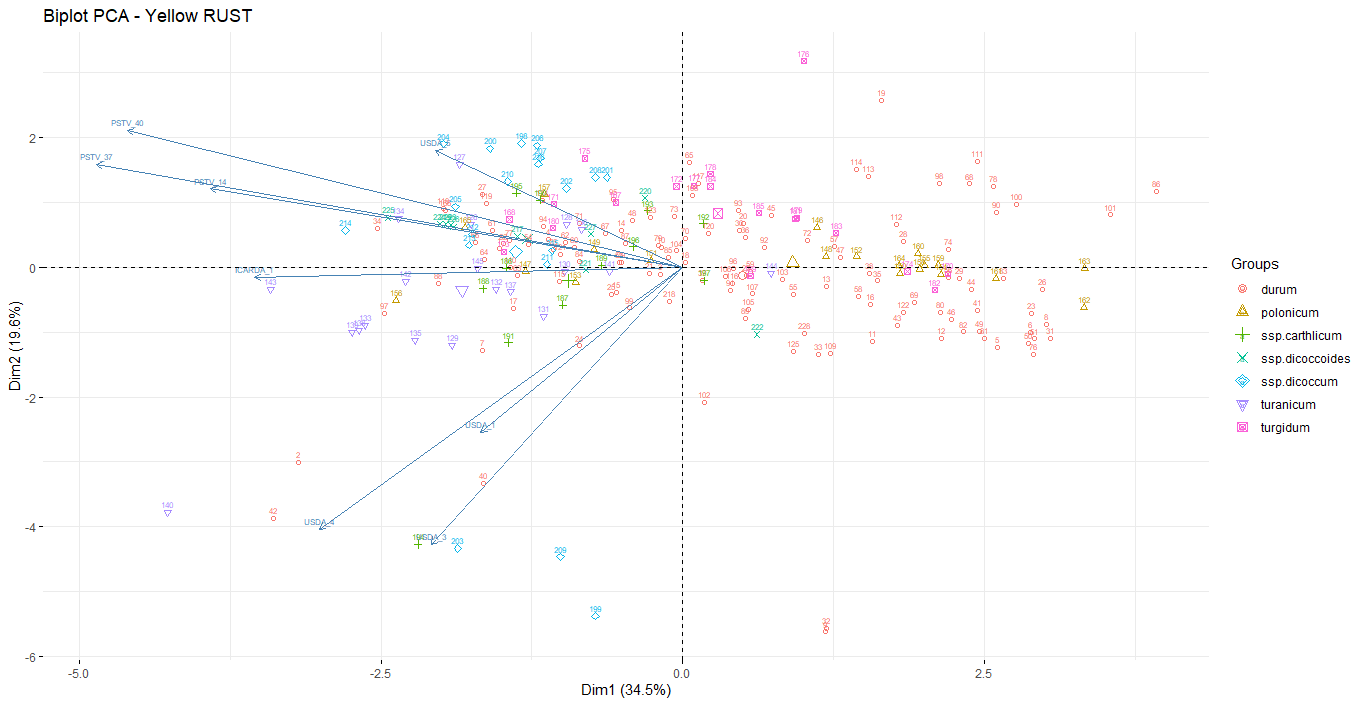
**

**SM 1 - Fig14.** PCA Biplot of leaf rust seedling and adult experiments.


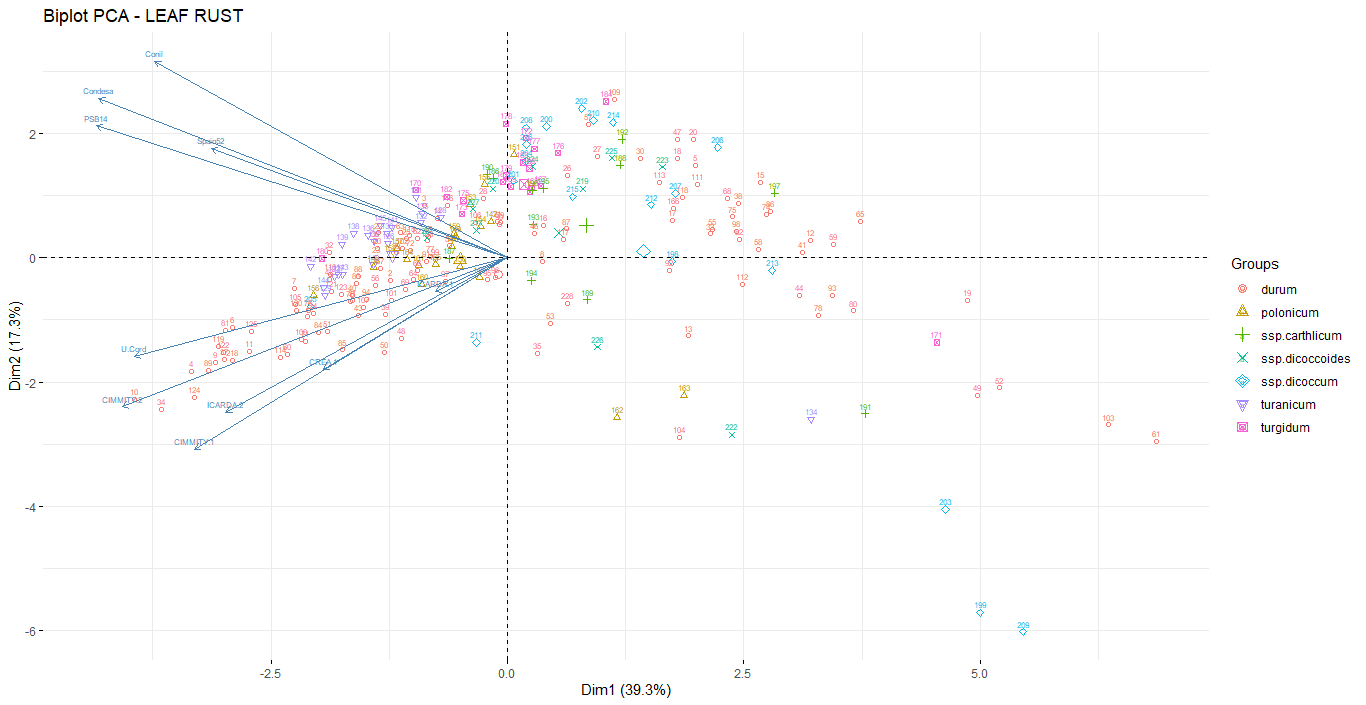

Supplement: Supplementary file 1 [file DataSheet_1.docx]
