# Supplementary material for: Genome-wide association study of common resistance to rust species in tetraploid wheat
Source: Front Plant Sci. 2024 Jan 3;14:1290643. doi: 10.3389/fpls.2023.1290643 (PMC10792004; doi:10.3389/fpls.2023.1290643)
Supplement: Supplementary file 2 [file DataSheet_2.docx]

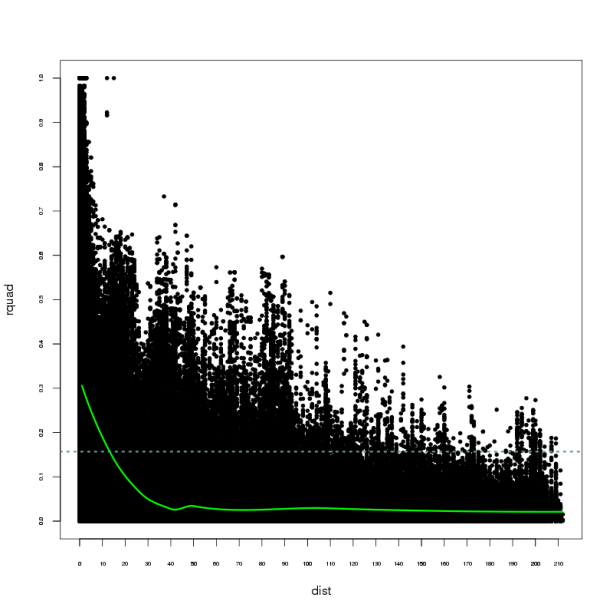

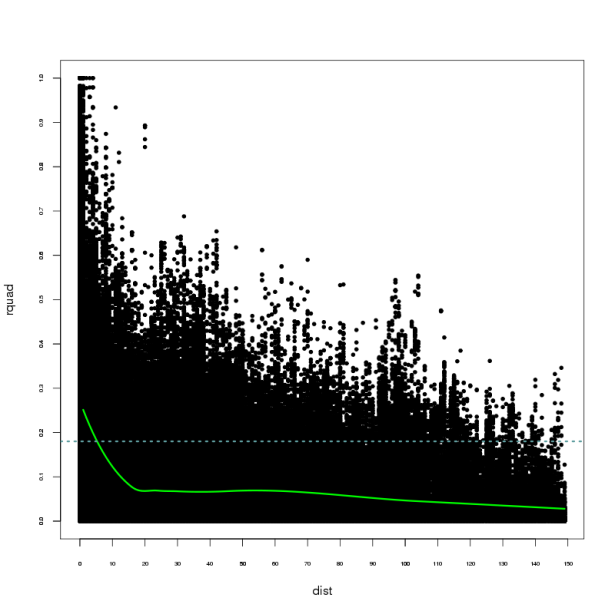


1A

2A


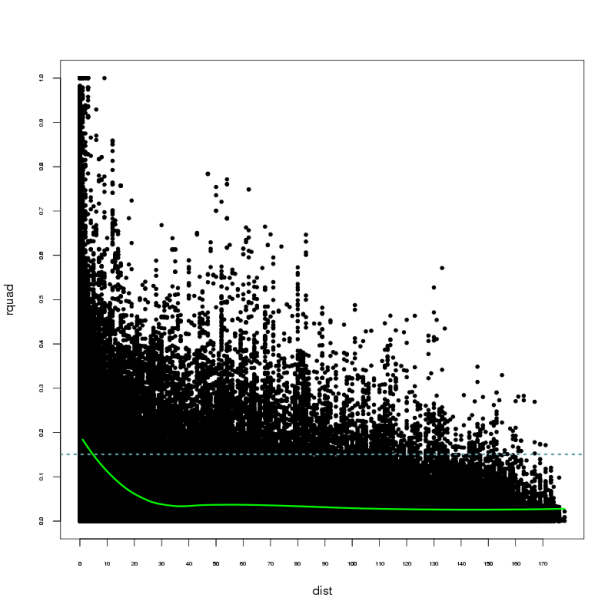

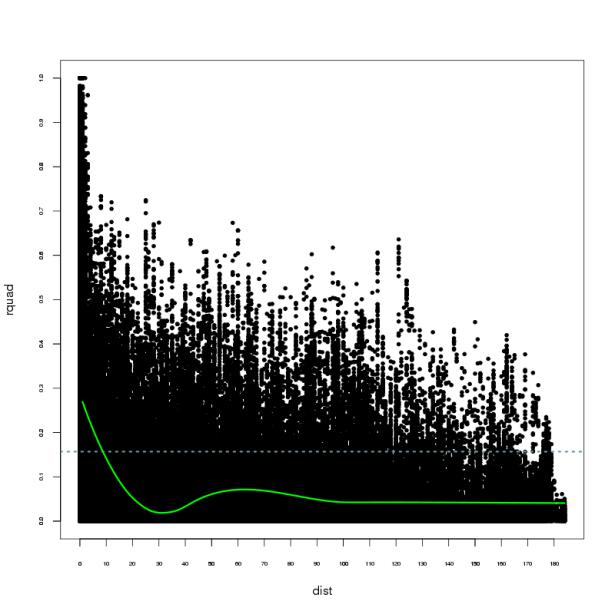


3A

4A


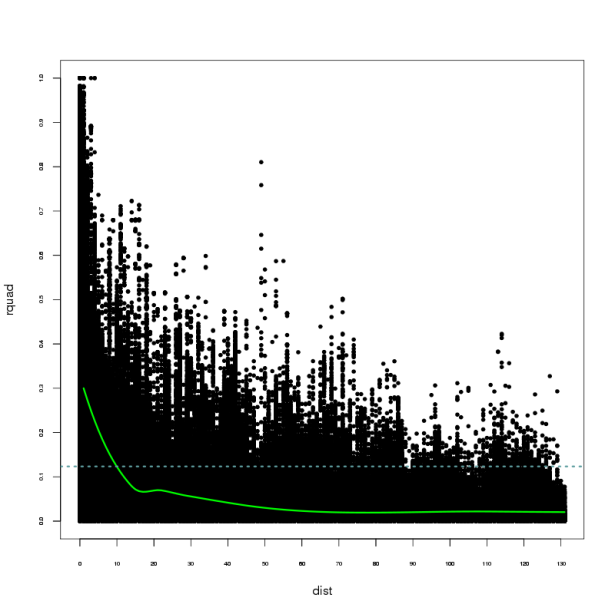

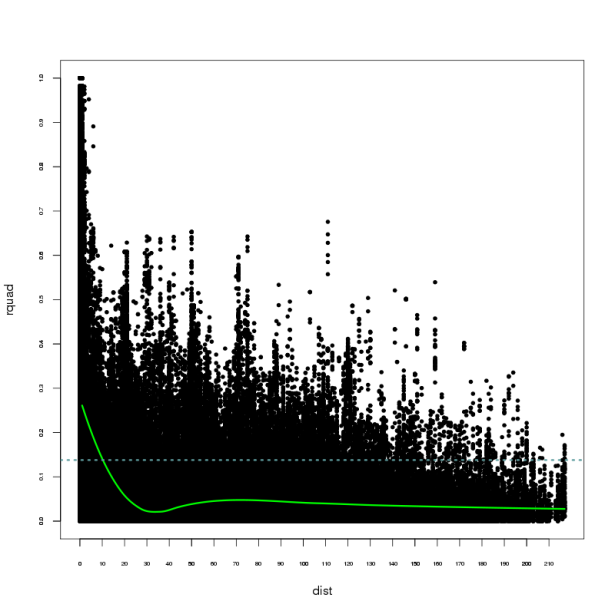


6A

5A


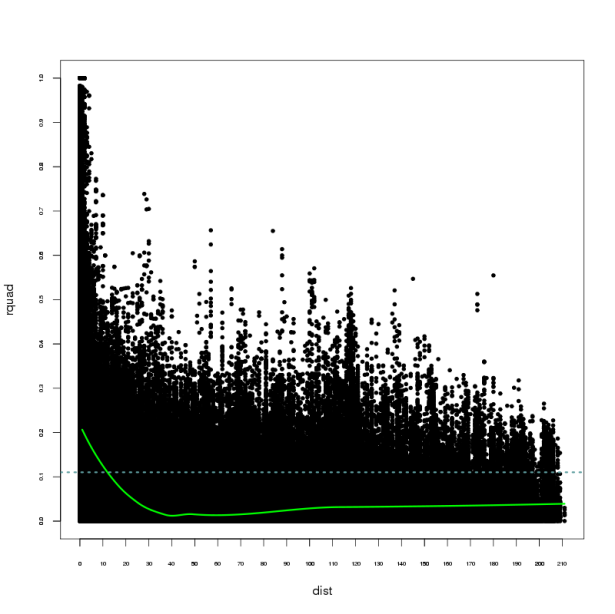


**SM2.1** Overview of the LD parameter r^2^ for the single chromosome of genome A considering the whole collection. The scatterplots show the distributions of the LD parameter r^2^ according to the genetic distance. The horizontal line indicates the 95% percentile of the distribution of the unlinked r^2^, which gives the critical value of r^2^.

7A


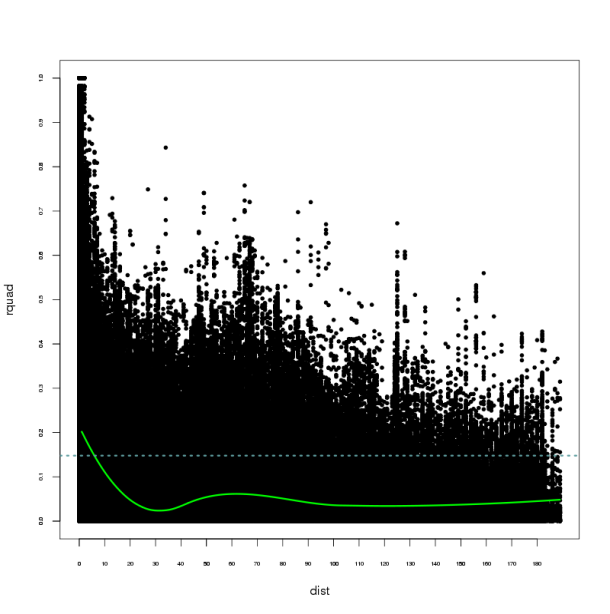

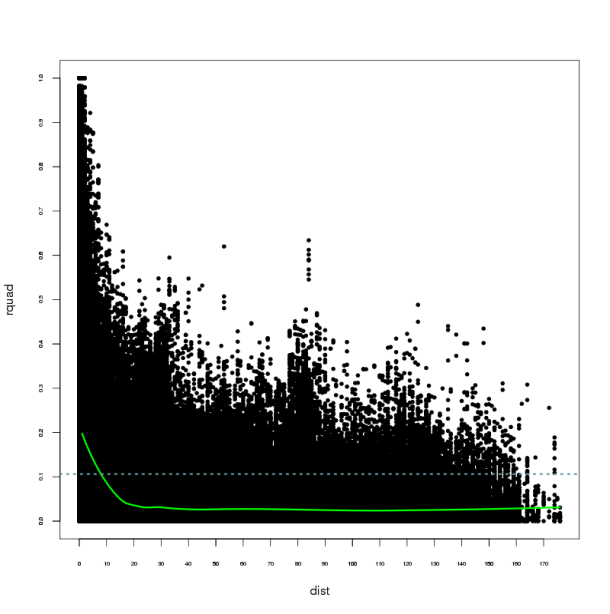


2B

1B


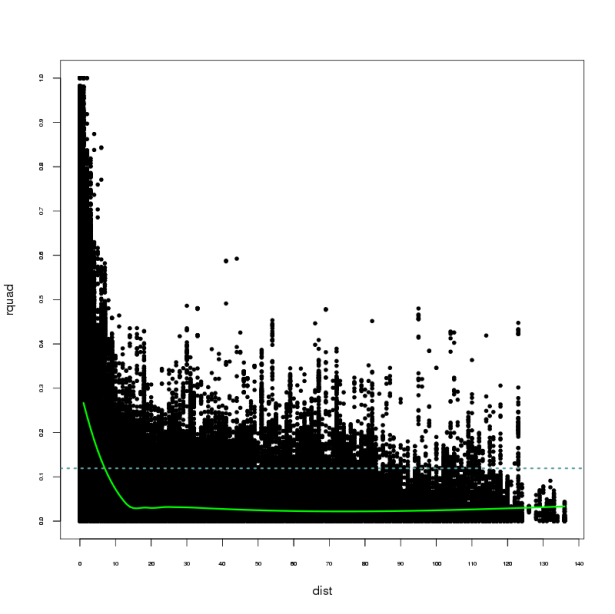

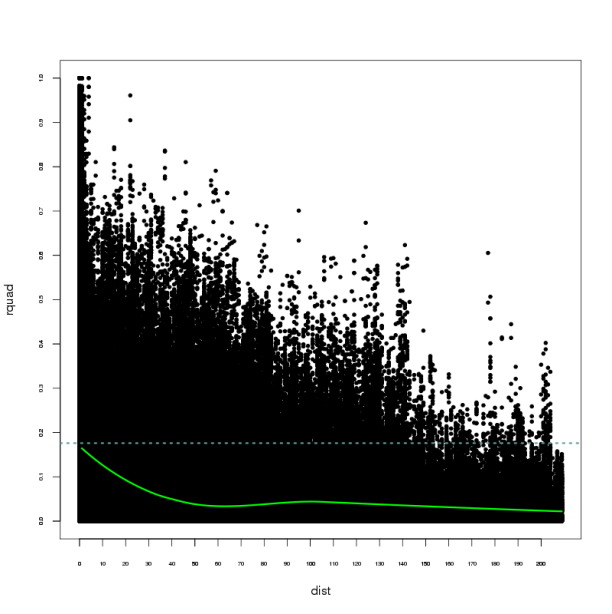


3B

4B


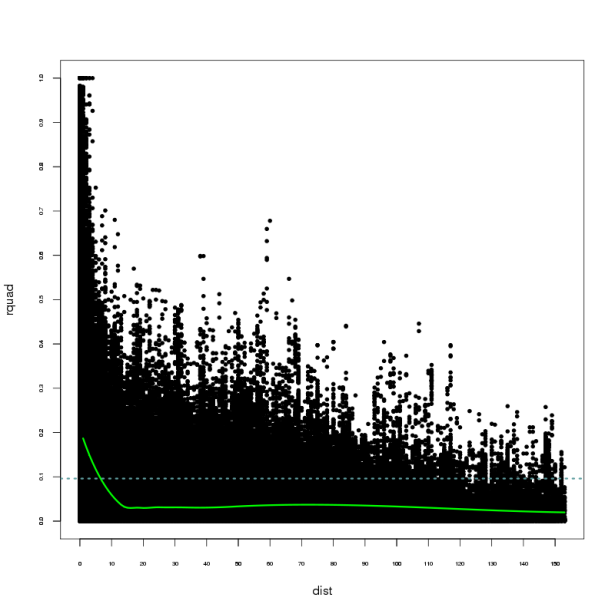

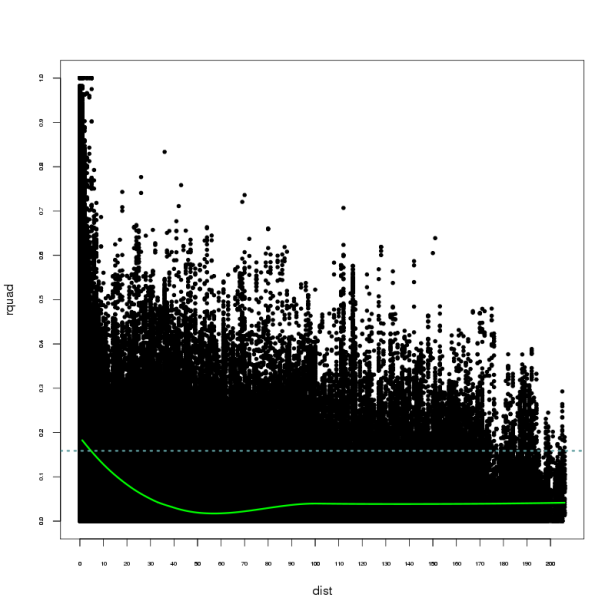


6B

5B


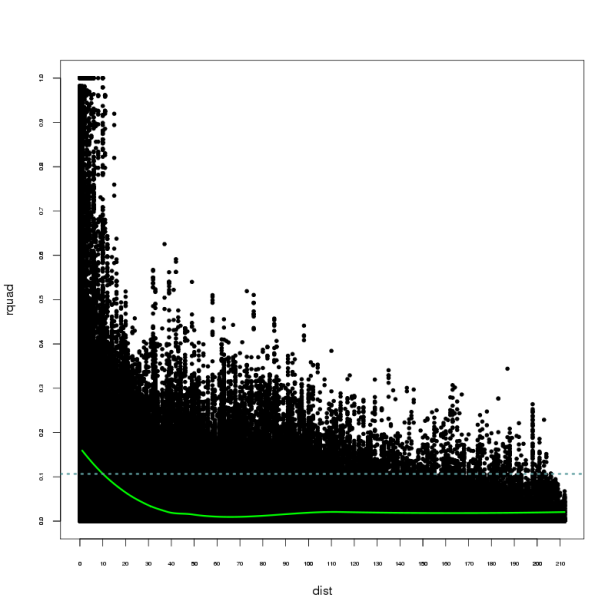


**SM2.2** Overview of the LD parameter r^2^ for the single chromosome of genome B considering the whole collection. The scatterplots show the distributions of the LD parameter r^2^ according to the genetic distance. The horizontal line indicates the 95% percentile of the distribution of the unlinked r^2^, which gives the critical value of r^2^.

7B
