# Supplementary material for: Genome-wide association study of common resistance to rust species in tetraploid wheat
Source: Front Plant Sci. 2024 Jan 3;14:1290643. doi: 10.3389/fpls.2023.1290643 (PMC10792004; doi:10.3389/fpls.2023.1290643)
Supplement: Supplementary file 4 [file DataSheet_4.docx]

**
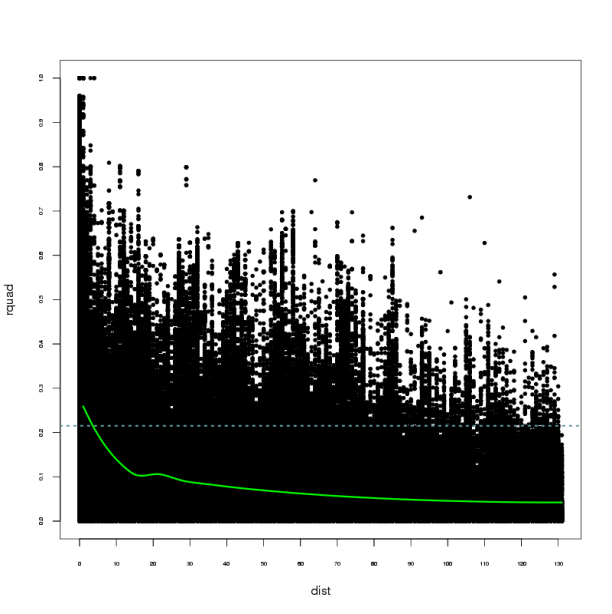

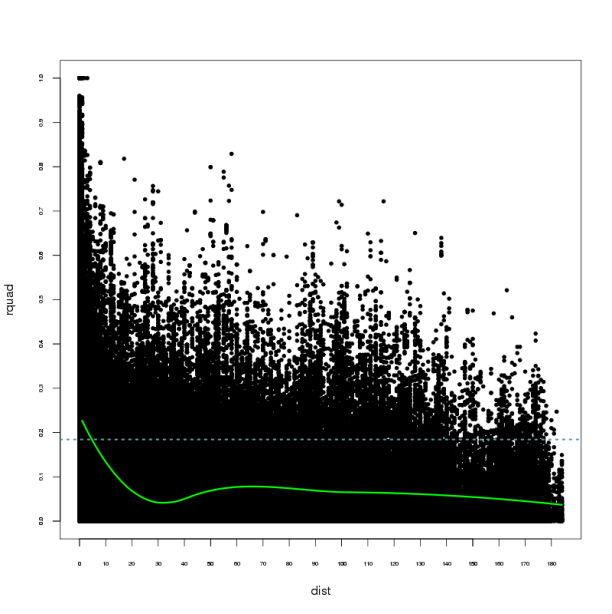

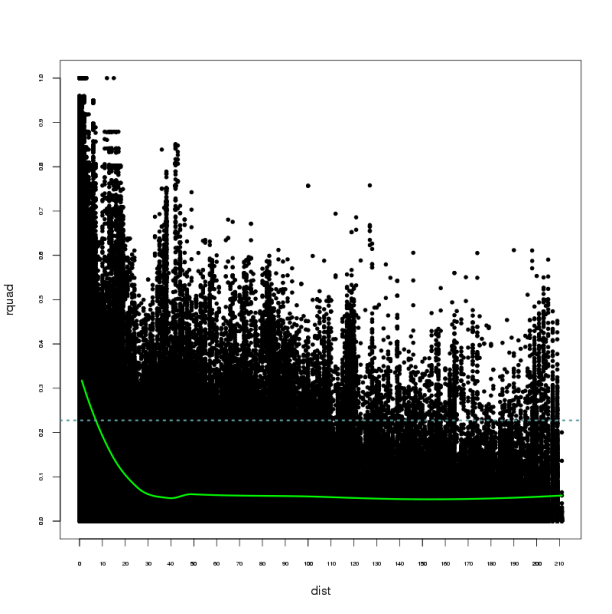

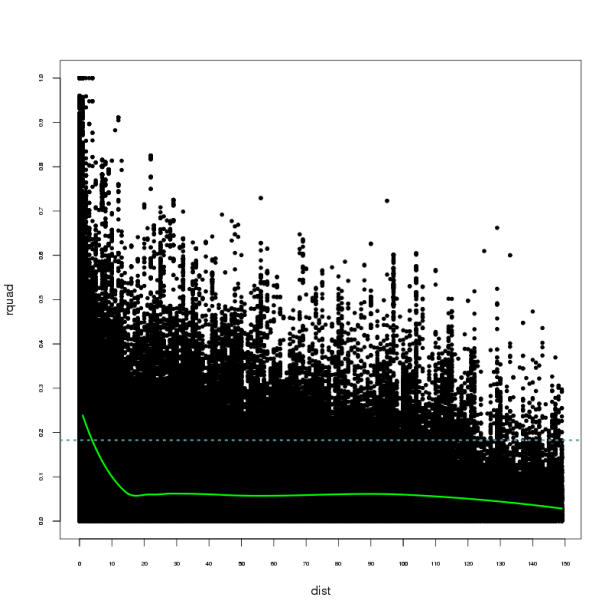

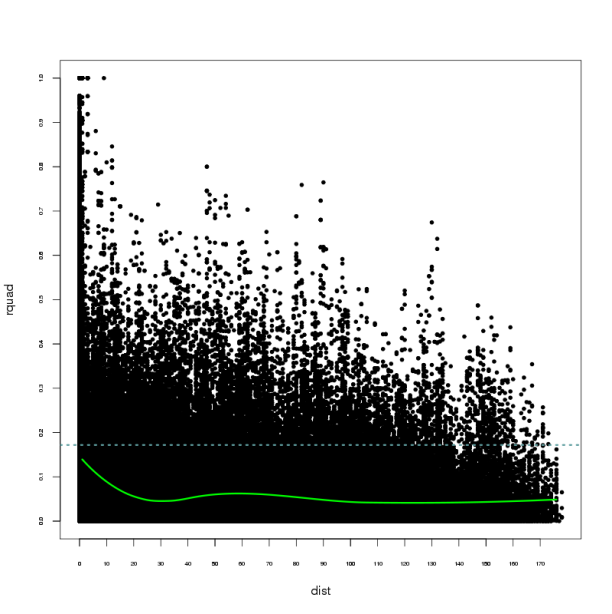

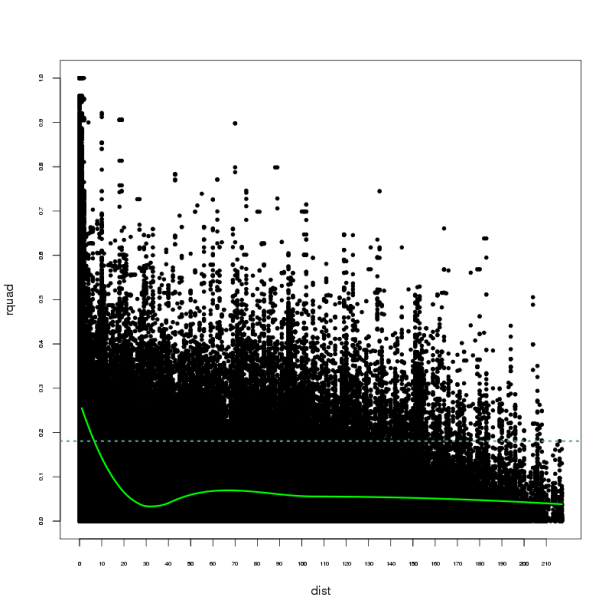

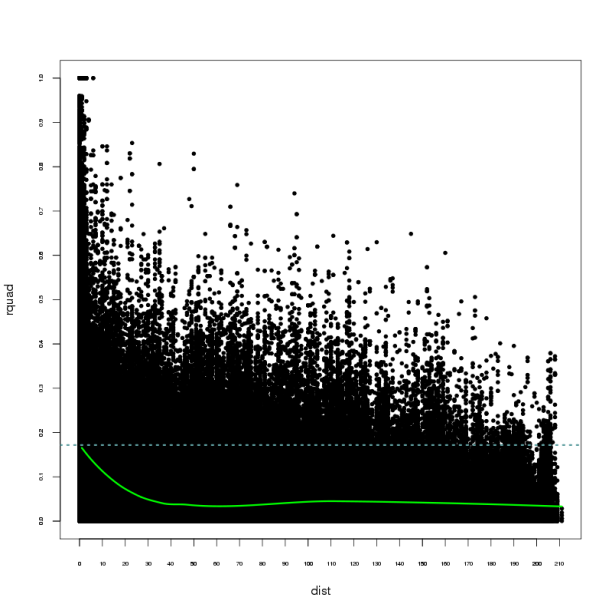
**

1A

3A

5A

7A

6A

2A

4A

**SM4.1** Overview of the LD parameter r^2^ for the single chromosome of genome A considering the Q2 group. The scatterplots show the distributions of the LD parameter r^2^ according to the genetic distance. The horizontal line indicates the 95% percentile of the distribution of the unlinked r^2^, which gives the critical value of r^2^.

**
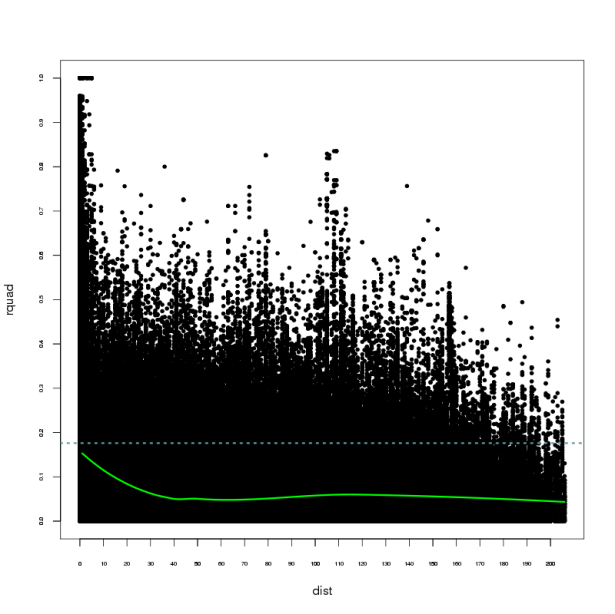

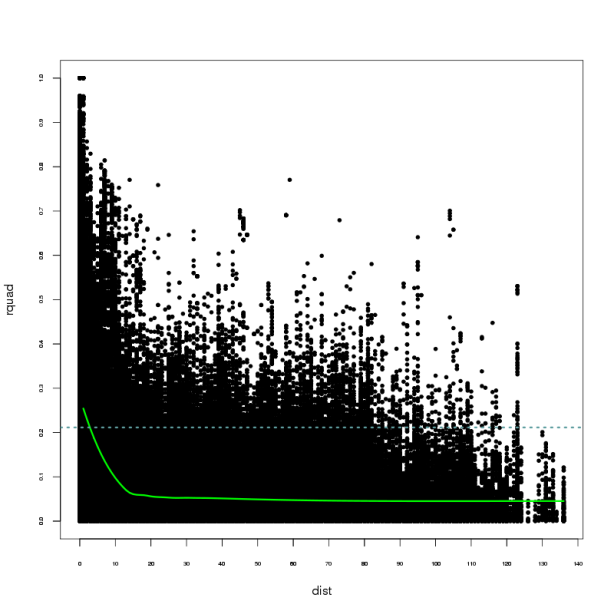

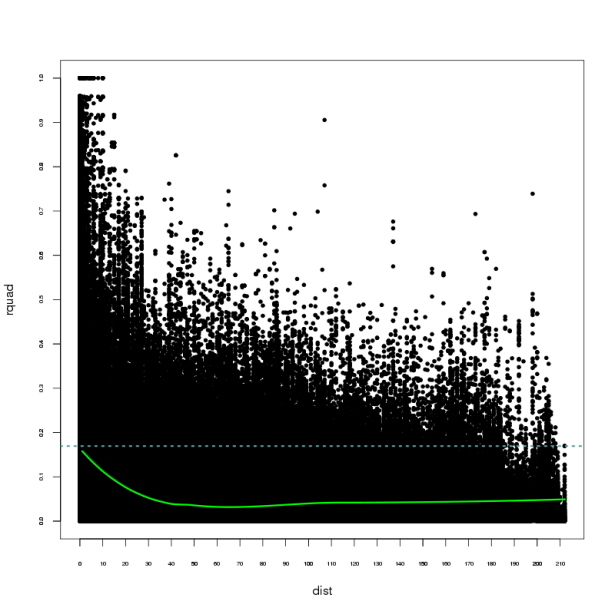

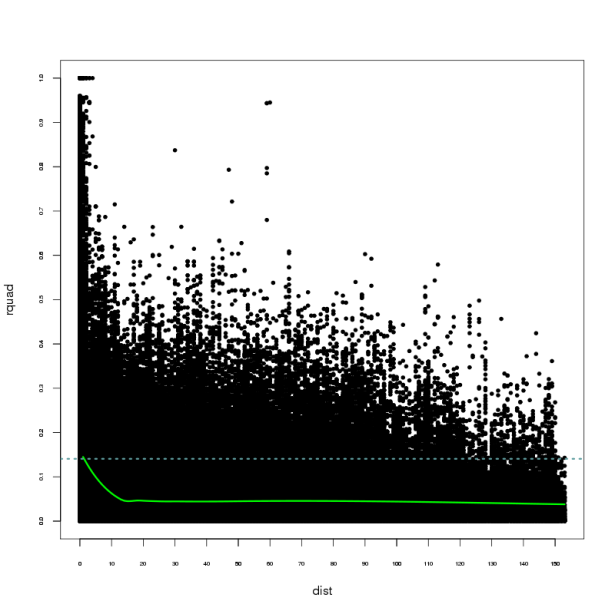

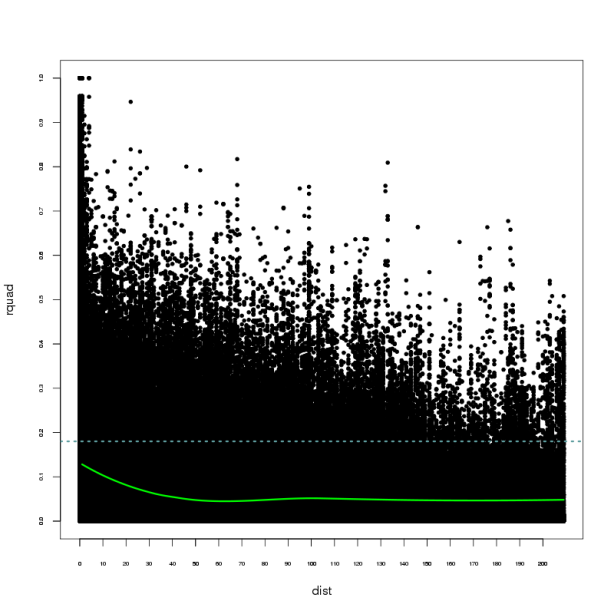

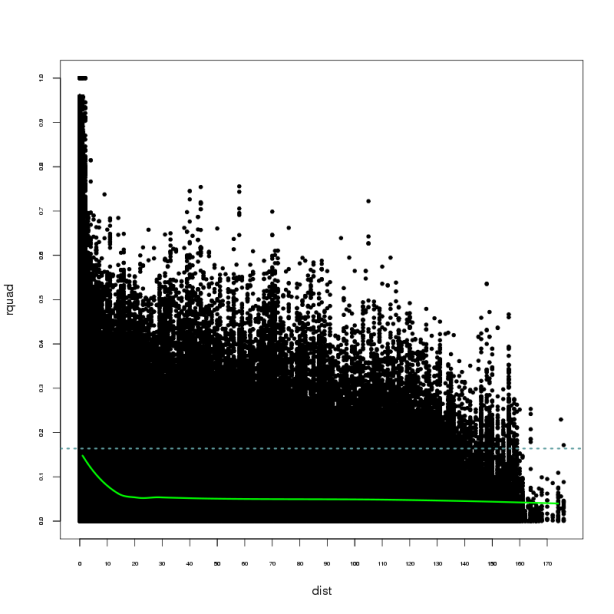

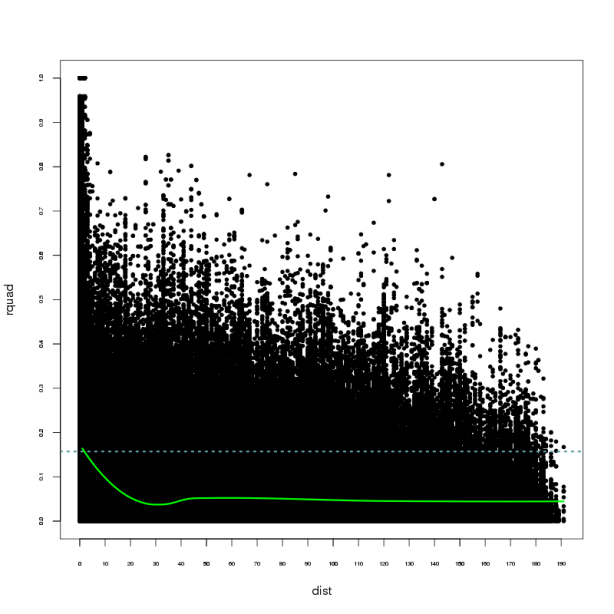
**

5B

6B

2B

4B

1B

7B

3B

**SM4.2** Overview of the LD parameter r^2^ for the single chromosome of genome B considering the Q2 group. The scatterplots show the distributions of the LD parameter r^2^ according to the genetic distance. The horizontal line indicates the 95% percentile of the distribution of the unlinked r^2^, which gives the critical value of r^2^.
