# Supplementary material for: Genome-wide association study of common resistance to rust species in tetraploid wheat
Source: Front Plant Sci. 2024 Jan 3;14:1290643. doi: 10.3389/fpls.2023.1290643 (PMC10792004; doi:10.3389/fpls.2023.1290643)
Supplement: Supplementary file 5 [file DataSheet_5.docx]

**SM5-a** QQ-plot determined by MLM+K model for Stem rust experiment (University of Minnesota, St. Paul Campus) at adult plant stage for a) Whole collection, b) Q2.


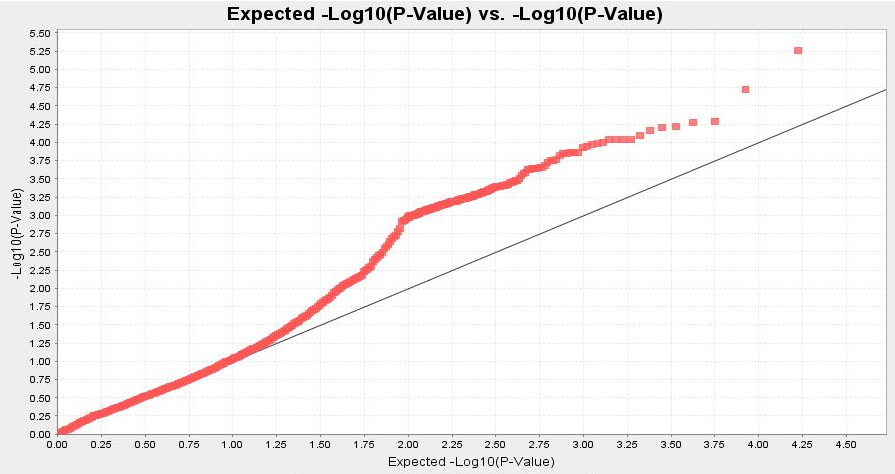


K

a


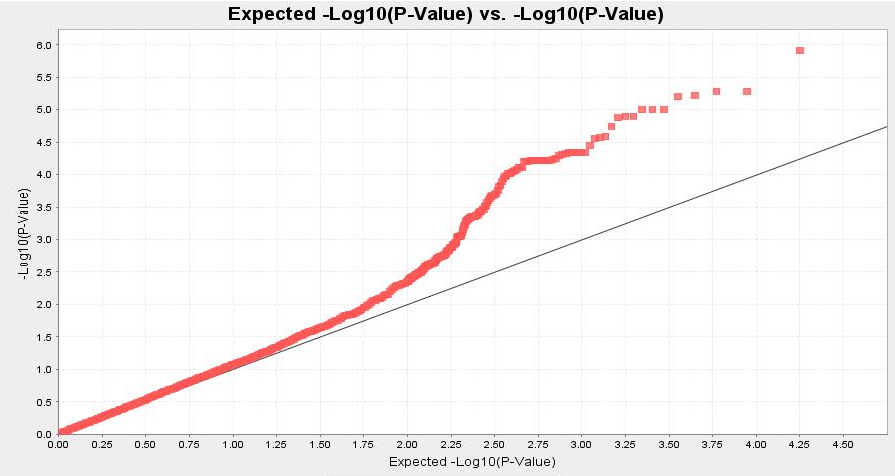


K

b

**SM5-b** QQ-plot determined by MLM+K statistical model for Stem rust seedling experiments (6 races) for a) Whole collection, b) Durum sub-sample, c) Q2.


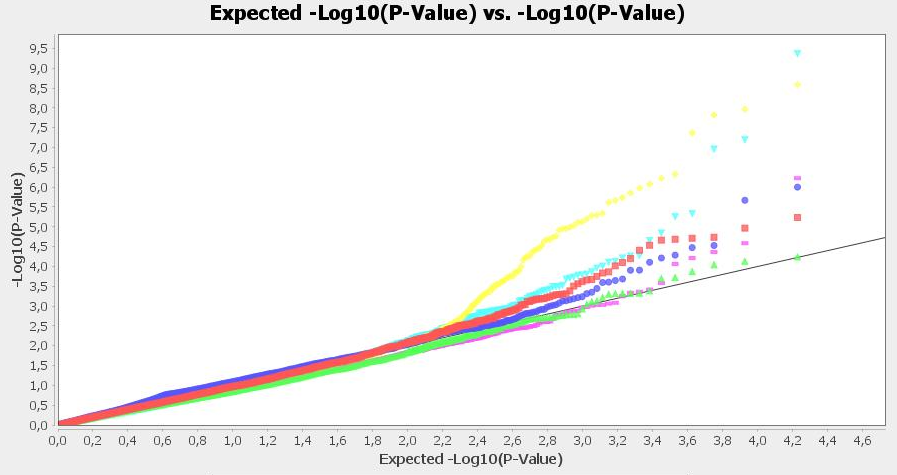


d (JRCQC) e (TKTTF) b (TPMKC) c (TRTTF) a (TTTTF) f (TTKSK)

a


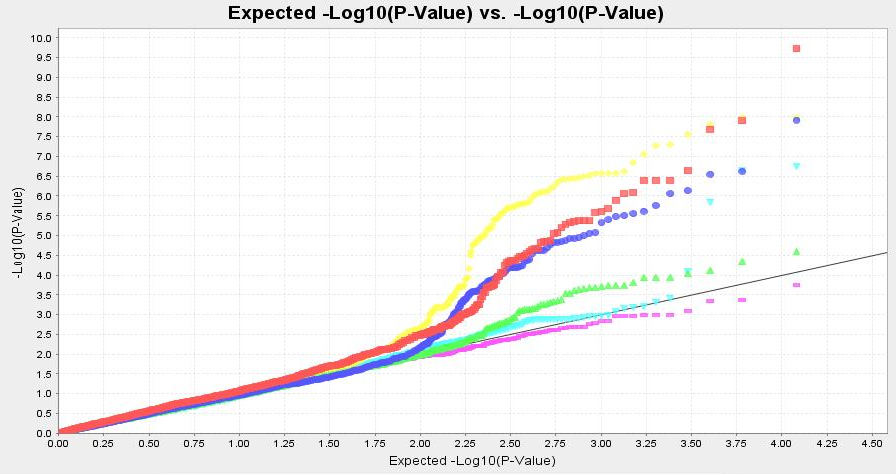


d (JRCQC) e (TKTTF) b (TPMKC) c (TRTTF) a (TTTTF) f (TTKSK)

b


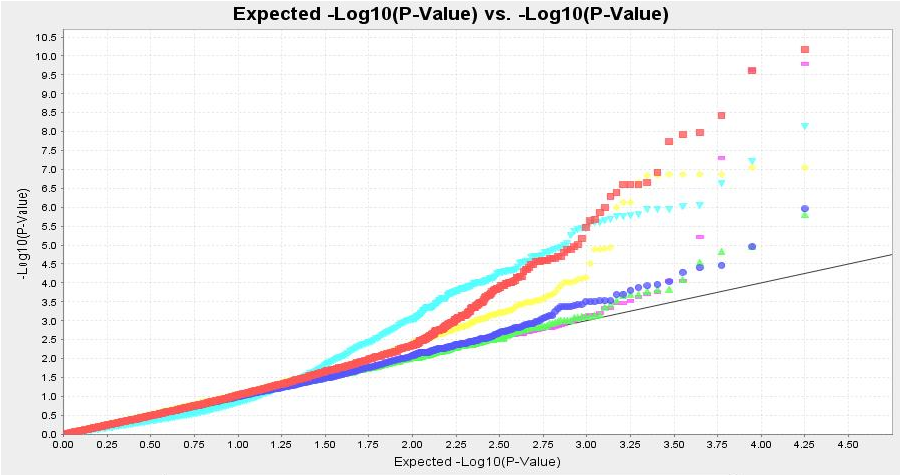


d (JRCQC) e (TKTTF) b (TPMKC) c (TRTTF) a (TTTTF) f (TTKSK)

c

**SM5-c** QQ-plot determined by MLM+K model for 7 field experiments of Leaf rust at the adult stage for a) Whole collection, b) Durum sub-sample, c) Q2, d) MLM+K+Q (2cM K4) for Q2 (experiments I and F).


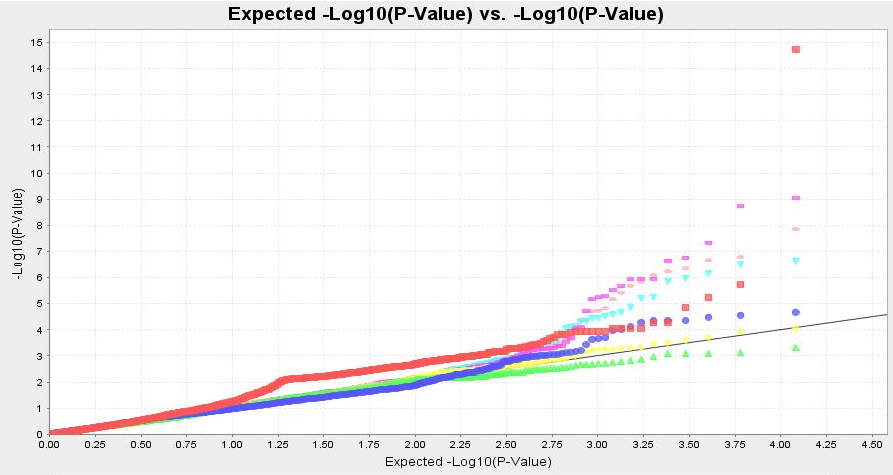


E G I L N F P

b


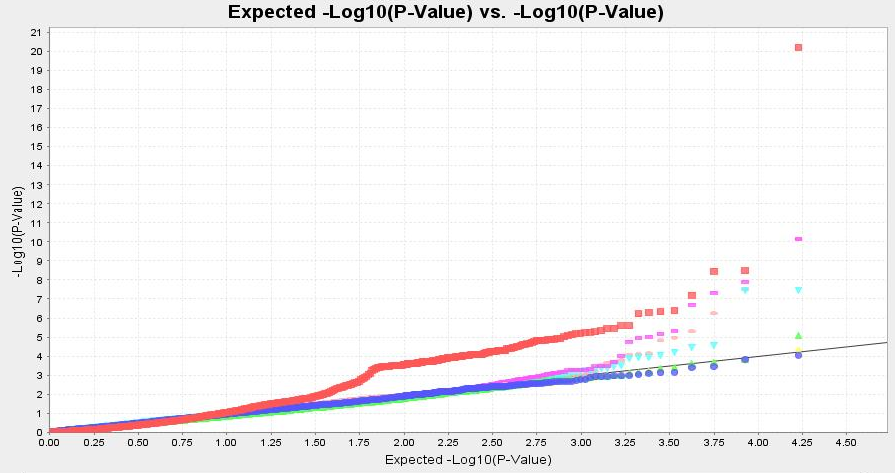


E G I L N F P

a


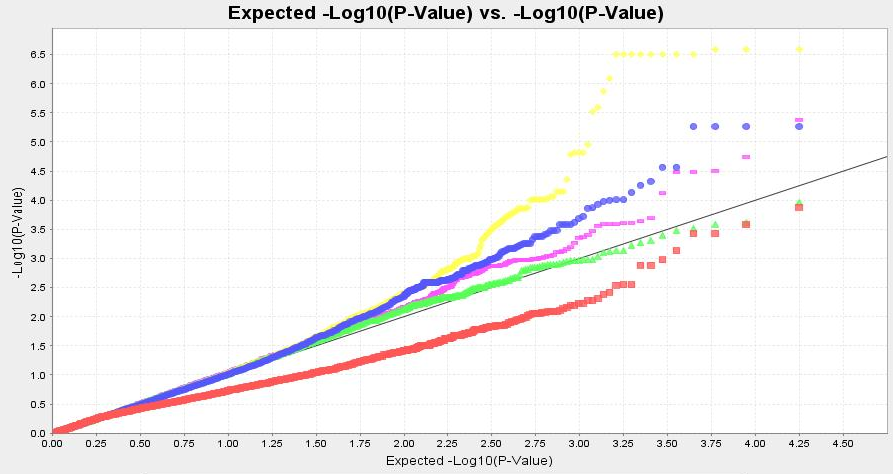


E G L N P

c


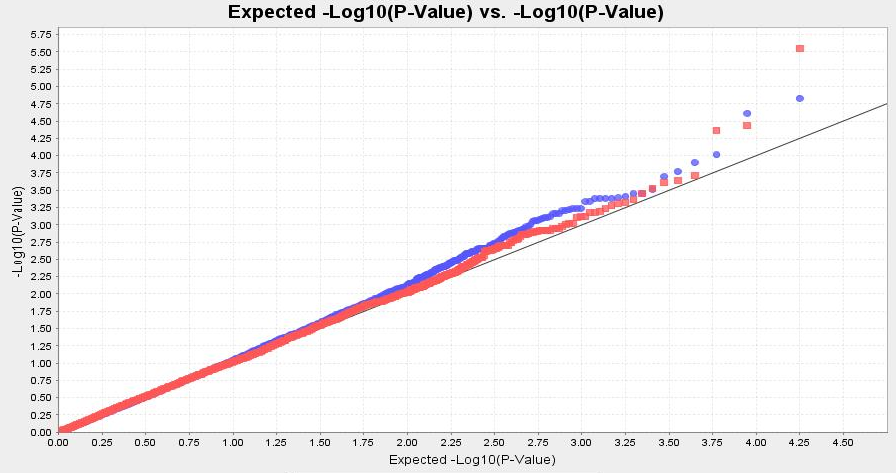


I F

d

**SM5-d** QQ-plot determined by MLM+K model for Leaf rust experiments at the seedling stage for a) Whole collection, b) Durum sub-sample, c) Q2.


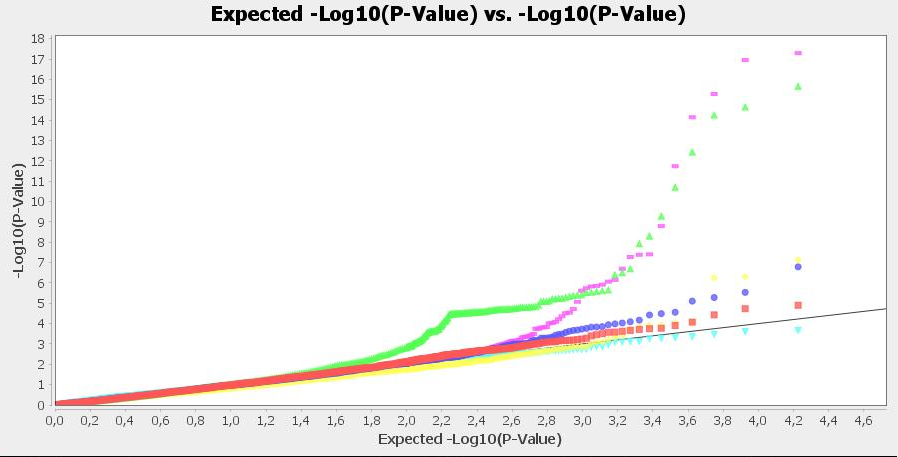


l (PSB14) m (Spain 5-2) n (V. Condesa -IT ) n (v. Condesa -DS) o (Conil -IT) o (Conil -DS)

a


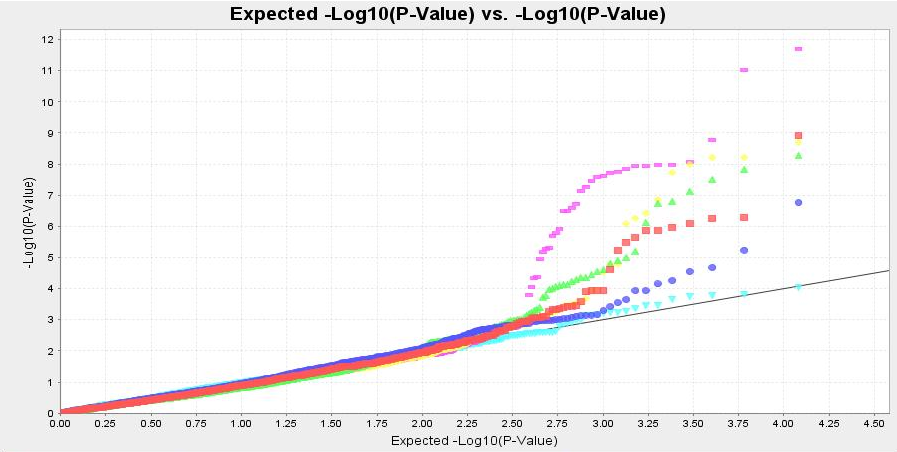


l (PSB14) m (Spain 5-2) n (V. Condesa -IT ) n (v. Condesa -DS) o (Conil -IT) o (Conil -DS)

b


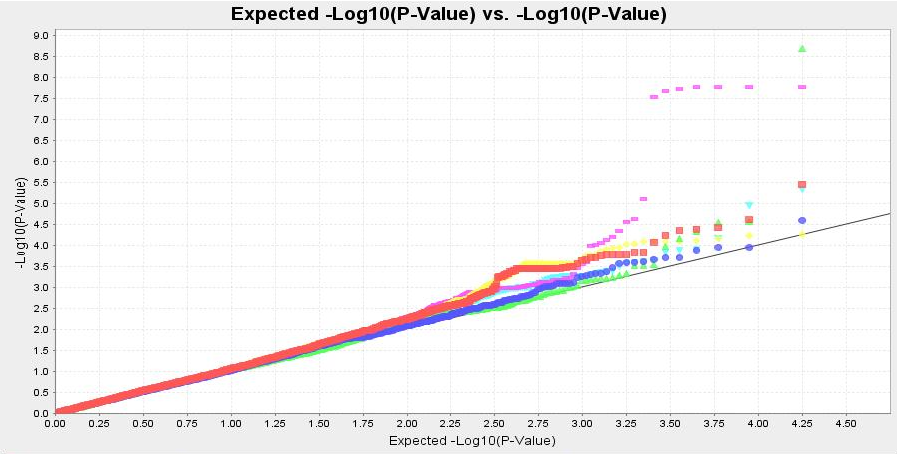


l (PSB14) m (Spain 5-2) n (V. Condesa -IT ) n (v. Condesa -DS) o (Conil -IT) o (Conil -DS)

c

**SM5-e** QQ-plot determined by MLM+K model for Stripe rust experiments at the adult plant stage (11 evaluations) for a) Whole collection, b) Durum sub-sample, c) Q2 and MLM+K+Q (K6 r=0.8).


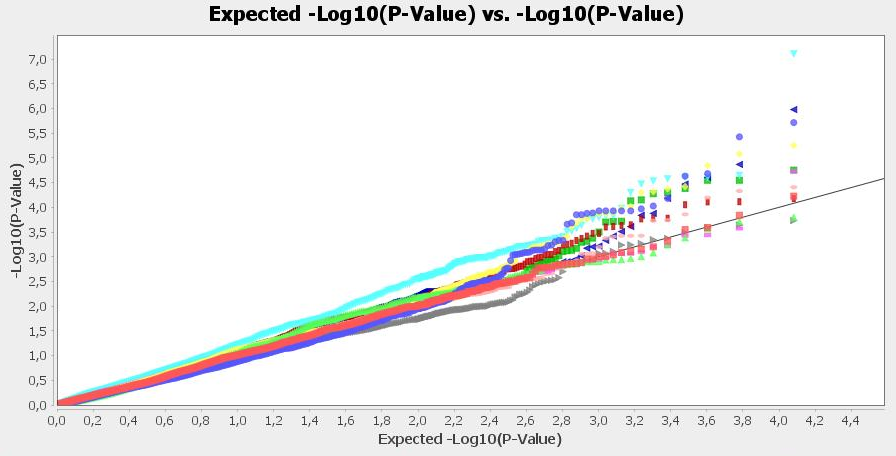


A (IT) A (DS) B (IT) B (DS) C (IT) C (DS) D H M O J

b


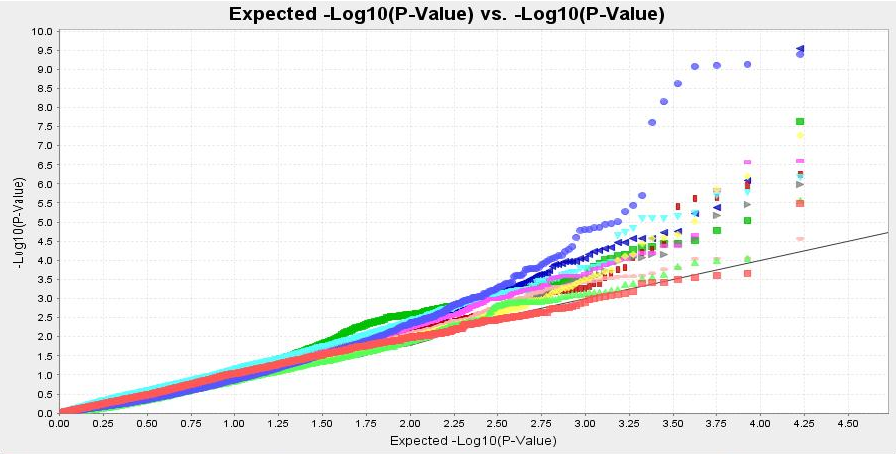


A (IT) A (DS) B (IT) B (DS) C (IT) C (DS) D H M O J

a


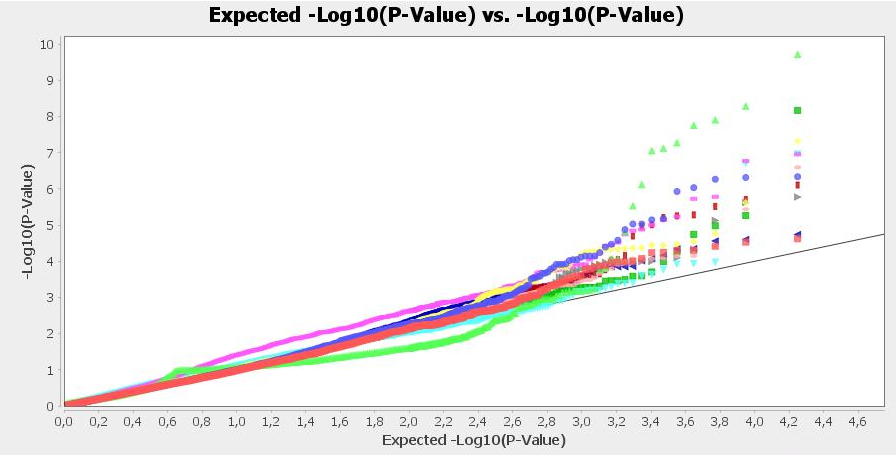


A (IT) A (DS) B (IT) B (DS) C (IT) C (DS) D H M O J

c

**SM5-f** QQ-plot determined by MLM+K model for Stripe rust seedling experiments (three races) for a) Whole collection, b) Durum sub-sample, c) MLM+K+Q (2cM K8) Q2.


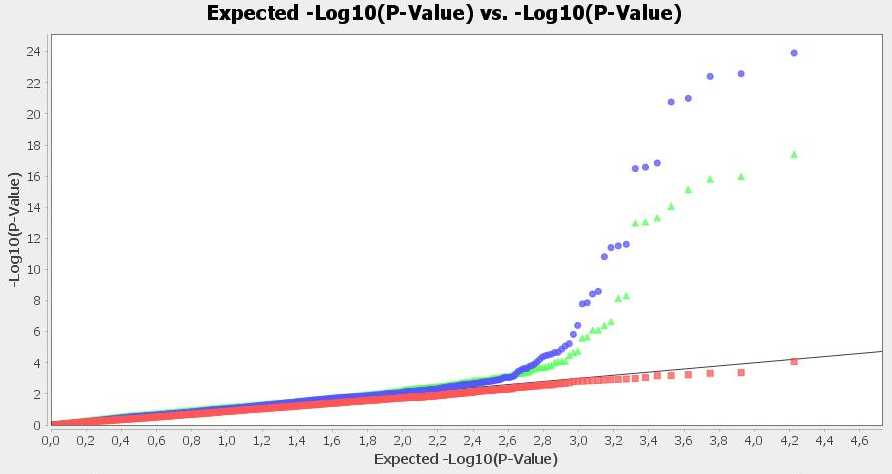


g (Pstv-14) h (Pstv-37) i (Pstv-40)

a


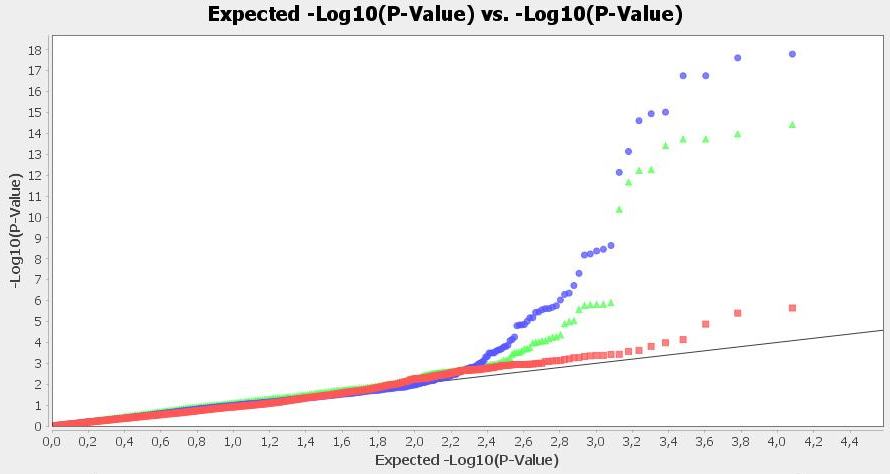


g (Pstv-14) h (Pstv-37) i (Pstv-40)

b


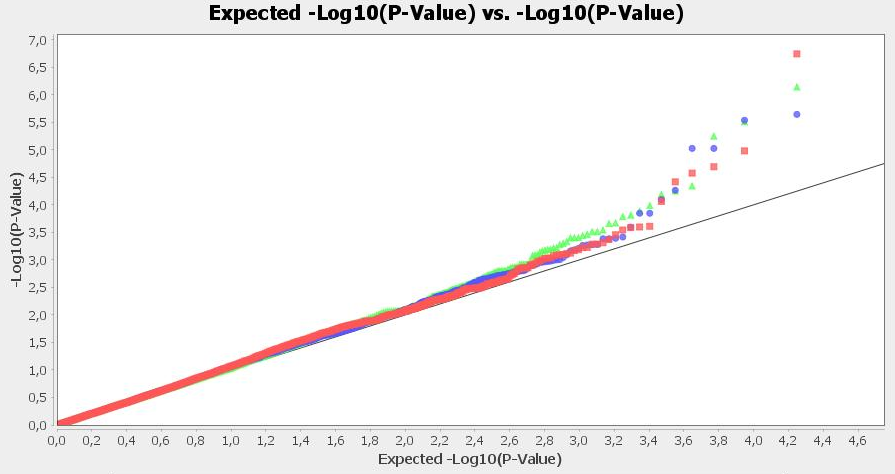


g (Pstv-14) h (Pstv-37) i (Pstv-40)

c
